# Obtaining Human Ischemic Stroke Gene Expression Biomarkers from Animal Models: A Cross-species Validation Study

**DOI:** 10.1038/srep29693

**Published:** 2016-07-13

**Authors:** Yingying Wang, Yunpeng Cai

**Affiliations:** 1 Research Center for Biomedical Information Technology Shenzhen Institutes of Advanced Technologies, Chinese Academy of Sciences, Shenzhen, China

## Abstract

Recent studies have revealed the systematic altering of gene expression in human peripheral blood during the early stages of ischemic stroke, which suggests a new potential approach for the rapid diagnosis or prediction of stroke onset. Nevertheless, due to the difficulties of collecting human samples during proper disease stages, related studies are rather restricted. Many studies have instead been performed on manipulated animal models for investigating the regulation patterns of biomarkers during different stroke stages. An important inquiry is how well the findings of animal models can be replicated in human cases. Here, a method is proposed based on PageRank scores of miRNA-mRNA interaction network to select ischemic stroke biomarkers derived from rat brain samples, and biomarkers are validated with two human peripheral blood gene expression datasets. Hierarchical clustering results revealed that the achieved biomarkers clearly separate the blood gene expression of stroke patients and healthy people. Literature searches and functional analyses further validated the biological significance of these biomarkers. Compared to the traditional methods, such as differential expression, the proposed approach is more stable and accurate in detecting cross-species biomarkers with biological relevance, thereby suggesting an efficient approach of re-using gene biomarkers obtained from animal-model studies for human diseases.

With the advent of molecular biotechnology, investigations on the molecular mechanism of cerebrovascular accidents are garnering increasing attention^1^,^2^,^3^,^4^,^5^,^6^,^7^,^8^. Through microarray analysis, recent studies have revealed that the genomic profile of human peripheral blood cells rapidly respond to cerebrovascular system damage^9^,^10^. Within the first 3 to 5 hours of stroke onset, a pervasive alternation of the gene expression profile can be observed from peripheral blood cells^9^, which affects multiple types of blood cells, including monocytes, polymorphonuclear leukocytes, neutrophils, and platelets^11^. Further investigations indicate that the impacts of cerebrovascular damage on blood gene expressions are propagated through various pathways, including inflammatory and immune response, cell growth and differentiation, hypoxia, vascular repair, and altered cerebral microenvironment^12^. Moreover, the patterns of genomic alternation during stroke are clearly distinguishable from other types of vascular diseases, such as myocardial infarction^13^. In addition to mRNA, former studies have also shown that many miRNAs were dysregulated in the brain and blood tissue of rodent ischemic stroke models^14^,^15^,^16^ by binding to their targets. Thus, investigating the gene regulation process of peripheral blood cells not only aids in exploring the molecular dynamics and physiological details during stroke development, but it also provides a promising approach for the etiology, pathology, early diagnosis, prognosis, and even prevention of the disease.

Nevertheless, sample collection poses a severe challenge to in-depth studies of human stroke genomics. Due to the suddenness of stroke onset, it is difficult to capture the blood sample of patients at the desired stage. Furthermore, for ethical reasons, it is essentially impossible to deliberately control the clinical status of a patient to observe the corresponding gene expression changes. Accordingly, rather than human subjects, animal models are then employed by many studies to infer the genomic mechanism of stroke and discover potential molecular biomarkers^17^,^18^,^19^,^20^,^21^,^22^,^23^,^24^,^25^. With animal models, researchers are given greater freedom to probe the physiological and molecular changes in various organs, such as brain tissues^17^, rather than in only blood cells. Furthermore, through animal models, researchers are able to obtain a more comprehensive knowledge about vascular pathophysiology after stroke onset through different methods, such as gene regulation analyses^18^, erythropoietin- induced changes analyses^19^, biological factors (such as age)^20^, pathways^21^, stroke-related processes (such as neuronal injury)^22^, reactive astrocytes^23^, immune responses to dying neurons, glia and vessels^24^, cell survival and death, and tissue repair and functional recovery^25^. Although there has been significant progress made in stroke genomics with the aid of animal model studies, an essential question is raised for this type of study, which is to what degree can the conclusions generated from animal models be replicated in human cases? Most researchers usually use the biomarkers directly obtained from animal models and carry out experiments on human subjects in order to verify them. This approach, however, has a high chance of failure, even when the selected biomarkers are mutually expressed in humans and animals alike. Because the gene regulation mechanism involves a complex network interaction between genes, the fact that humans and animals share a mutual gene does not necessarily imply that this gene has equal informative value on the same disease. Therefore, a more sophisticated method should be developed so as to efficiently explore the results obtained in animal experiments and achieve gene biomarker models that are applicable to human cases.

In this paper, an approach to extract gene biomarkers and prediction models from the results of animal model experiments that can be reliably replicated on human subjects is proposed. The basic idea is to make use of the gene interaction information revealed in the development of the disease and identify hub genes that are essential in forming the gene interaction systems. In contrast to traditional differential expression analysis, this approach is more robust across platforms because hub genes are key components of the gene regulatory network that reflects the full picture of gene interaction, which is more stable across species as compared to individual genes. The technology is further applied to build a gene diagnosis model from the animal gene expression data and validate it on several human gene expression datasets of stroke patients. Due to the large number of differentially expressed mRNAs and the small number of training samples, it is impractical to construct a complete gene interaction network. In taking advantage of the fact that miRNA has been discovered to play important regulatory roles in stroke development^26^, this study solely investigates miRNA-mRNA interactions for identifying mRNA biomarkers. A parallel miRNA-mRNA expression profile from rat brain tissue is used to construct a network based on negative correlation calculation. The PageRank^27^ algorithm is then used to calculate the importance of nodes including miRNAs and mRNAs and rank them in order so as to choose the important nodes as the featured biomarkers. Two datasets of human blood mRNA expression profiles are then used to test the biomarkers. The results demonstrate that most of these markers are related to stroke, and they could clearly cluster different conditions. These results confirm the important value of animal stroke genomics studies on human stroke cases, and at the same time, the results emphasize the necessity of developing cross-species, cross-platform analysis technologies for this type of research.

## Materials and Methods

### Microarray datasets

The study involved two types of microarray datasets. The mRNA and miRNA expression profiles from manipulated rat samples were used for identifying stroke gene biomarkers. Then, two human mRNA expression profiles from healthy and stroke patients were used to validate the biomarkers discovered in the first stage.

### Acute ischemic stroke expression profiles in rat model as the training set

Parallel miRNA-mRNA expression profiles of permanent focal ischemia that was induced by permanent occlusion of the left middle cerebral artery (MCA) using a sub-temporal approach^28^ in an *in vivo* male wistar rats model (GSE25676) were downloaded from NCBI GEO^29^,^30^.

The experimental group was treated as follows: (1) anesthetized rats with ketamine (75 mg/kg, intraperitoneal) and xylazine (10 mg/kg, intraperitoneal) and (2) exposed the MCA through a subtemporal craniectomy and cauterized it from the point proximal to its origin to the point where it intersected the inferior cerebral vein. Ischemic injury samples with i.c.v. injection of 80% DMSO and 30 mM ZM447439 in 80% DMSO were named as the vehicle and treatment groups, respectively. The sham group was operated in the same way as the experimental group, just without the MCA occlusion. The RNA samples were collected from the right cortex of rats at 2 time-points (8-hour and 24-hour) for 3 experimental conditions (sham, vehicle, and treatment).

The dataset (GSE25676) was composed of two datasets: mRNA (GSE23651) and miRNA (GSE25556) expression profiles. The mRNA expression profiles (GSE23651) were performed on an Illumina ratRef-12 v1.0 expression beadchip (GPL6101) with 22,524 probes for six conditions (the combination of 2 time-points and 3 experimental conditions) including Sham-8 h (n = 4), Vehicle-8 h (n = 4), Treatment-8 h (n = 4), Sham-24 h (n = 4), Vehicle-24 h (n = 4), and Treatment-24 h (n = 4). The miRNA expression profiles (GSE25556) were performed on a miRCURY LNA microRNA Array, 5th generation - hsa, mmu & rno (GPL11241) with 361 probes for the same samples as the mRNA expression profiles.

### Ischemic stroke expression profiles in human blood as the test sets

Two ischemic stroke mRNA expression profiles were downloaded from the NCBI GEO database that involved ischemic stroke patients in either the acute or recovery stage, along with healthy controls. The GSE16561 dataset contained the mRNA expression profiles of 39 acute ischemic stroke patients and 24 control subjects with the total RNA extracted from the whole blood and analyzed on the platform of Illumina HumanRef-8 v3.0 expression beadchip (GPL6883)^31^. The stroke patients were all older than 18 years old with an MRI diagnosed as ischemic stroke, and the controls were non-stroke neurologically-healthy people. There were 24,426 probes that could be mapped to 18,491 genes. The GSE22255 dataset contained the mRNA expression profiles of 20 ischemic stroke patients that had only suffered one stroke episode that had occurred at least 6 months before the blood collection and 20 sex- and age-matched control subjects that did not have a family history of stroke. The total RNAs were extracted from PBMCs and were analyzed on the platform of the Affymetrix Human Genome U133 Plus 2.0 Array (GPL570)^32^.There were 54,676 probes that could be mapped to 20,284 genes.

### Framework of Data Analyses

**Figure 1:**
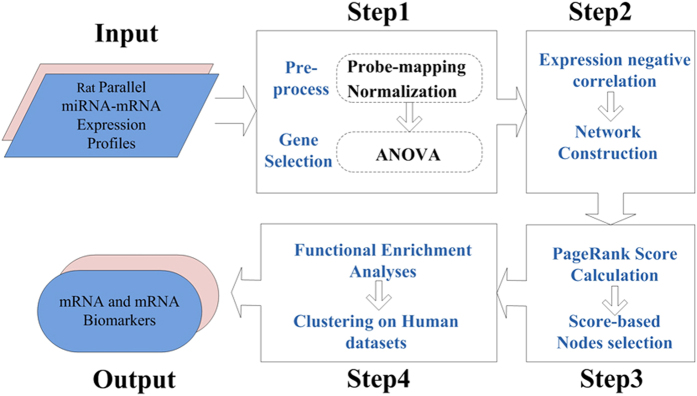
Framework of this study.

The systematical analyses were performed according to the following steps (see Fig. 1 for details):

#### Step 1 Data Pre-process and Gene Selection

In order to carry out cross-species and cross-platform analyses, gene mapping was performed on the three datasets and 11,400 genes were found to be mutually available across all three mRNA expression datasets and were used in the subsequent analyses. The expression value of a gene was the mean value of all of its probe sets because a gene may have a few transcripts due to alternative splicing or alternative promoters. For rat miRNA data, the 361 probes in the raw miRNA expression profiles were mapped to 279 mature miRNAs. Data normalization and gene selection were then carried out on the rat dataset. The mRNA dataset was normalized using median normalization, whereas the miRNA dataset was normalized using the global lowess regression algorithm. ANOVA was then performed for each gene mRNA expression profile among the 6 experiment conditions. Genes with a p-value of less than or equal to 0.05 were chosen as the differential expressed genes.

#### Step 2: Network Construction

It is been widely accepted that miRNAs normally regulate their targets in a negative way, which means their expression values are negatively correlated^33^,^34^. The parallel microRNA-mRNA rat expression profile data with rat brain samples were used to construct the negatively correlated network. All miRNAs and mRNAs with an ANOVA p-value less than or equal to 0.05 were chosen to calculate the expression relationships.

#### Step 3: Node Selection

The PageRank algorithm was used to find the important nodes in the network generated in Step1. The miRNAs and mRNAs were ranked separately, and the top ranked ones were chosen as the featured biomarkers.

#### Step 4: Human Data Validation

Two human blood mRNA expression profiles were used to test the classification ability of the mRNA biomarkers selected in Step2 based on the rat stroke model.

The details of Step 2 through Step 4 are described in the following subsections.

### Network construction

After gene selection, 8885 mRNAs were chosen as differentially expressed genes (with p < 0.05), and they were used to construct a miRNA-mRNA interaction network by connecting them with all 279 miRNA profiles. The correlation score between each miRNA-mRNA pair was calculated using the Pearson’s correlation coefficient *r*, which is defined as the covariance of the two molecular variables divided by the product of their standard deviations as follows:


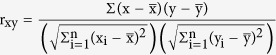


where *x* represents one miRNA and y represents one mRNA. The range of *r* was [−1, 1] with 0 indicating no correlation, −1 indicating a strong negative correlation, and 1 indicating a strong positive correlation. A statistical test was performed based on the Pearson’s product-moment correlation coefficient, and a *p*-value was given to show the significance of the r value. All the pairs with a *p*-value less than or equal to 0.05 and negative correlation scores (p < 0.05 and r < 0) were chosen to construct a weighted network. The nodes in the networks covered miRNAs and mRNAs, and each edge was weighted using the absolute value of the correlation score between the two nodes it connected.

It should be noted that, although only miRNA-mRNA links are considered in the network, due to the nature of statistical correlation, once a miRNA is involved in a signaling pathway, the mRNAs that directly interact with that miRNA as well as all of the mRNAs along the pathway will be assigned a score if the disturbances from other connections can be neglected. Thus, the method proposed in this paper will not only select genes that interact directly with many miRNAs, but it will also select hub genes that participate actively in various miRNA-mediated signaling pathways.

### Node selection

The R package ‘igraph’^35^ was used to perform the Google PageRank^27^ analyses on the network using the Pearson’s correlation coefficient as edge weights. The PageRank algorithm has been used by Google Search to measure and rank the importance of website pages in search results by treating page links as a network and each web page as a node. With the underlying assumption that more important nodes are likely to have more links from other nodes, the algorithm counts the number and quality of links to a target node to make a rough estimate of the importance of that node. Initially, all nodes in the network are assigned an equal score. Then, the score of each node is transferred to its outbound connected neighbors in a strength proportion to the connection weights in each of the iteration, until a stable state is met. The final score is used to measure the importance of a node. A detailed description of the algorithm is available in ref. 36 PageRank and similar methods have been successfully used in gene expression analyses in recent years, including signaling crosstalk identification^36^, clinical outcome prediction^37^, miRNA-mRNA prediction^38^, and so on. As compared to traditional approaches, the aforementioned methods exhibit certain advantages, such as robustness and the ability to find potential important biomarkers. In this study, this algorithm was used for the selection of mRNA biomarkers by measuring their importance in the miRNA-mRNA interaction network. PageRank was executed on the miRNA-mRNA bipartite network that was constructed in Step 2 described above. The miRNA and mRNA were ranked separately after each was assigned a score by calculation. The top-ranked mRNAs were chosen as biomarkers for validation (See Supplementary Table S1 for details). Due to the difficulties of achieving high-quality miRNA profiling with existing technology, miRNA biomarkers were not used for validation. Nevertheless, the top-rank miRNAs are listed in Supplementary Table S2 and their biological significance was validated on human subjects through literature search in the Result section.

### Human Data Validation

Because the behavior of informative biomarkers may vary across species or disease stages even when the underlying mechanism is identical, it is not sufficient to directly test the cross-species replicates of the selected biomarkers using a prediction model built from animal samples. In order to validate the performance of the obtained gene biomarkers on human subjects, hierarchical clustering was carried out on the two human validation datasets in order to test whether these biomarkers could clearly separate healthy and stroke-afflicted people. The two test sets (IS expression profiles in human blood) were normalized using median normalization. The average-link hierarchical clustering was performed using function ‘heatmap.2’ in R package ‘gplots’ using the selected top-ranked mRNA biomarkers. In the ideal case, stroke patients and healthy people would be clustered into two distinct clusters. The number of incorrectly clustered samples was used to evaluate the quality of the biomarkers.

### Functional Annotation

In order to understand the biological role of the obtained gene biomarkers, functional annotation analyses were performed on the top-ranked mRNAs using the DAVID functional annotation tool^39^,^40^. (DAVID Bioinformatics Resources 6.7, NIAID/NIH) based on integrated resources including disease, functional categories, Gene Ontology^41^,^42^, pathways, and so on. In addition to providing annotation query results, DAVID also performed a modified Fisher exact test to verify the statistical significance of the resulting function terms using the whole human genome as the background. A p-value was then assigned for each annotation term. By default, DAVID adopted a p-value threshold of <0.1 and a hit count threshold of > = 2 to include an annotation term in the results. Moreover, function annotation clustering was carried out using fuzzy heuristic clustering to gather similar functions into clusters according to the degree of overlap between two annotations measured by the Kappa values. The geometric mean of the log-transformed p-values for all group members was used as the group enrichment score to measure the significance of the group.

## Results

### Selected Biomarkers and Validation

Table 1 lists the top 20 ranked mRNAs as selected by the PageRank scores according to the above described framework. The Student’s t-test results (p-values) between the stroke and healthy groups for each mRNA feature on the two human validation sets are also given. It can be seen that the vast majority of the selected genes remain highly informative on the human validation datasets, which confirms the idea that network analyses provide reliable results across species and microarray platforms. It can also be observed that all of the top 20 genes (and most of the top 100 genes) were consistently expressed in the stroke patients of the two validation sets. In addition, in the training samples, most of the top ranked genes were highly distinctive before and after the stroke, but they showed weak or no distinctions in the vehicle vs. treatment groups (p ~ 0.07 at 8 hour and p > 0.1 at 24 hours). Despite this, a significant number of genes can be found to be differentially expressed between the two stages. Taking into considering our criteria of selecting marker genes, this may indicate that the altering of gene expression patterns in the acute stage of stroke is more collective and systematic as compared with the recovery stage. Furthermore, the above observations indicate that some of the gene expression changes brought on by stroke onset may be permanent to the suffering patient. A complete list of the top 100 selected mRNA gene biomarkers and their description information is provided in Supplementary Table S1 in the appendix.

**Figure 2:**
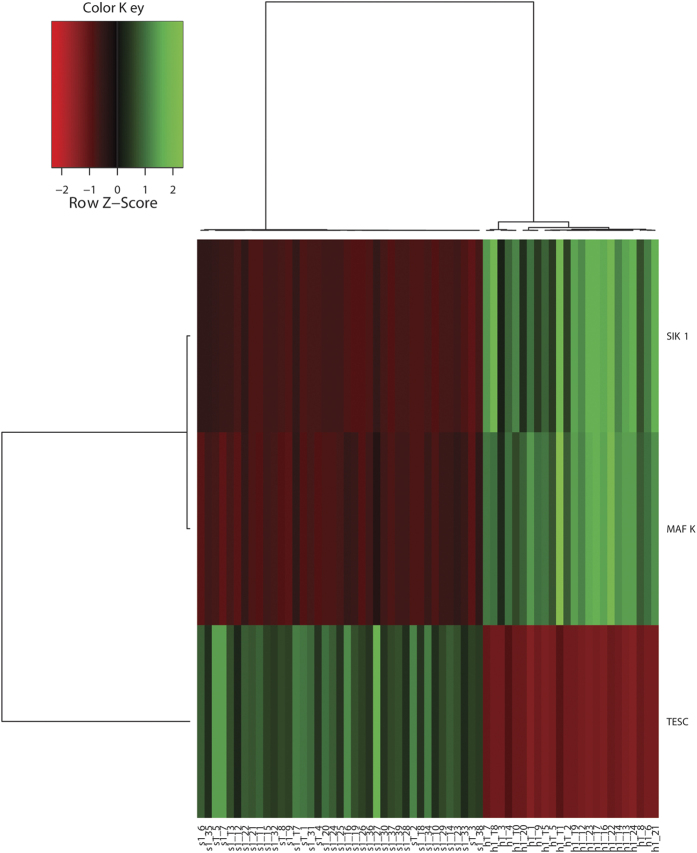
GSE16561 cluster analyses using top three selected features.

**Figure 3:**
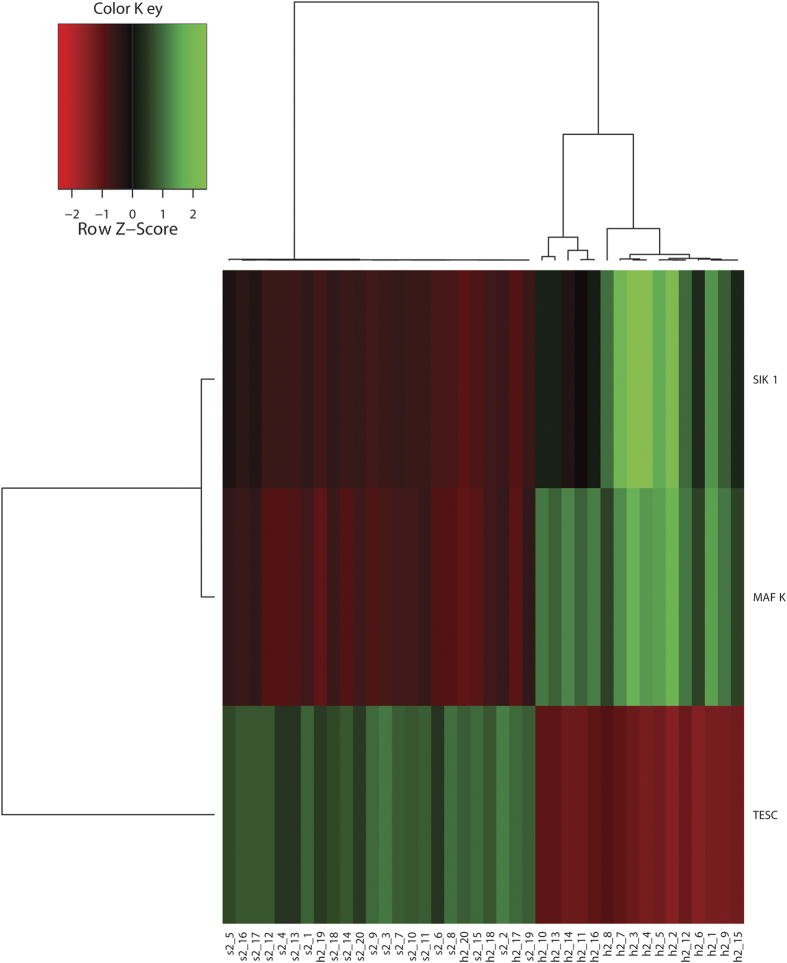
GSE22255 cluster analyses using top three selected features.

**Figure 4:**
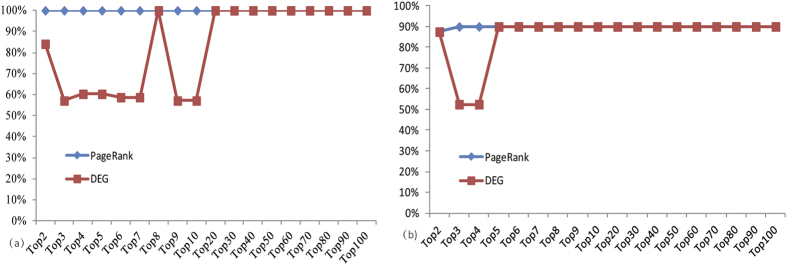
Comparison of clustering accuracy for both methods on test sets (**a**) GSE16561 (**b**) GSE22255.

To further demonstrate the gene selection performance, clustering analyses were carried out as proposed in the Methods section using the top three selected features of the two datasets. As is depicted in Figs 2 and 3, for dataset GSE16561, the top three features correctly clustered all patient and healthy samples, however, for dataset GSE22255, 90% accuracy was achieved with four healthy samples being misclassified into the patient group. The same separation can be observed using different numbers of marker genes (Fig. 4).

For comparison, a traditional differential expression (DE) analysis was also employed to select genes, and clustering analyses was performed using the top genes. Figure 4 depicts the comparison of the clustering accuracy for both methods on the two human validation datasets with varied number of selected biomarkers (from top 2 to top 100). It can be seen that the DE analysis can also pick out some genes that are replicable on human data; however, the clustering quality fluctuates with the different number of features used because the DE analysis produced a number of false gene biomarkers, which were uninformative on human data and degraded the overall performance. This further confirms our suggestion that network analyses are more reliable in selecting cross-platform biomarkers than traditional approaches.

### Functional analyses for top biomarkers

Among the top 100 genes and all of the 49 miRNAs in the network, 45 genes and 32 miRNAs were identified that have been reported to be involved in stoke through a literature search in NCBI PubMed and Google Scholar. As listed in Table 2, these biomarkers can be classified into the following groups: (1) biomarkers (genes) transcribed from stroke related genomic mutations (e.g., SNPs); (2) biomarkers (genes and miRNAs) involved in processes causing stroke onset and development (such as neuronal apoptosis, a rapid increase in excitatory neurotransmission, etc.); (3) biomarkers (genes and miRNAs) involved in biological processes accompanied with or after stroke (such as immune cell homeostasis, neuronal damage, etc.); (4) biomarkers (genes) involved in stroke recovery (such as the biosynthesis of N-acetylneuraminic acid, etc.); (5) potential stroke therapeutic targets (genes); (6) biomarkers (genes and miRNAs) that have been previously reported to be differentially expressed among stroke and healthy subjects or across stroke samples and subtypes (but without a clearly discovered mechanism); (7) biomarkers (genes and miRNAs) in the same family of known stroke-related markers, which can be inferred to also be likely stroke-related; and finally, (8) biomarkers (genes) interacting with/binding with/regulate stroke-related factors (such as p53, etc.). Furthermore, 15 of 45 genes and 24 of 32 miRNAs were validated on human subjects, 19 of 45 genes and 4 of 32 miRNAs were validated on animal models, and 2 of 45 genes and 2 of 32 miRNAs were validated both on human and animal models, using various techniques such as RT-PCR, western blot, microarray, immunohistochemistry, and so on. The broad overlap between the top-ranked biomarkers and existing literature further justifies that the present method is capable of picking out biomarkers that are replicable and biologically relevant. The relationships among the top-ranked miRNAs and mRNAs were examined using multiple miRNA target computational algorithm prediction results^43^,^44^. These top miRNAs were shown to regulate 23.35 top genes while the top genes were shown to be regulated by 11.44 miRNAs on average (see Supplementary Table S1 and Table S2 in the appendix for detail numbers of each top gene and miRNA). This indicates that the top biomarkers are closely related to each other which may explain the complex of IS to some degree.

Using DAVID, the gene functions of the top 100 genes were annotated and enrichment analyses were performed, as is shown in Supplementary Tables S3 and S4. The 100 enrichment terms were matched with a p-value < 0.1, and 12 genes were matched with known psychiatric diseases, which again justifies that the biomarkers discovered in this paper are hub genes that are actively involved in key biological processes. By summarizing the enrichment results, three core gene groups emerged (as is shown in Table 3), with most of the genes being previously known as stroke-related in literature. The first group contains 12 protein-kinase related genes (p = 0.0008), most of which are also involved in phosphorus metabolic processing (p = 0.0105) and ATP-binding (p = 0.0395). From among them, four genes (KCNH1, CAMK1G, CAMK2G, and CAMKK1) are related to calcium-calmodulin binding (p = 0.0002). This is consistent with previous findings that the dysregulation of protein kinase can be associated with stroke-induced injury^45^,^46^,^47^,^48^,^49^,^50^ and the finding that the inhibition of some protein kinases, especially calcium-calmodulin binding ones^51^,^52^ can be potential therapeutic targets^53^. The second group comprises 13 genes associated with cell cycles including proliferation, development, differentiation, and apoptosis following stress or stimulus responses, which reflects the compensatory reaction of the neural and cerebrovascular systems following stroke onset. Notably, the intracellular signal pathways (p < 0.08), including the MAPK pathway and the p53 pathway, clearly bridge the two gene groups, which validates previous studies that protein kinases mediating extracellular stimulations to intracellular responses play essential roles in ischemic pathologic conditions and that inhibitors of these pathways would be promising therapeutic agents for stroke treatment^54^. Also, the dual role of the MAPK pathway^55^ which promotes both post-stroke damage and recovering was validated by the above grouping results. The third group is composed of nine genes (with two also in group 2) concerning circadian rhythms (p < 0.0002) validated with previous literature^56^,^57^,^58^. Although the circadian rhythm of blood pressure is long known to be associate with stroke onset (e.g., ref. 59), the underlying molecular mechanism has not yet been well-studied, and the gene markers discovered here might provide valuable information for this topic.

More than half (53) of the top 100 genes were found to be related to phosphoprotein, which is significantly enriched compared with normal backgrounds (p < 0.002). Although previous studies have discovered that some kinds of phosphor-proteins (such as VASP) are involved with brain-blood barrier damage or neural protections following stroke and other cerebral diseases^60^, the pervasive correlation between the stroke marker genes and phosphoproteins suggests that more investigations are merited regarding the role of phosphoproteins in stroke onset and development.

## Discussion

Recently, there has been a growing interest in investigating the genetic and genomics factors of cerebrovascular and cardiovascular diseases. Genetic factors are suspected to contribute greatly to the onset of stroke since traditional vascular risk factors, such as hypertension, cigarette smoking and diabetes mellitus may account for only about 30% of the population-attributable risk of IS. However, being an acquired disease, the pathology of how genetic risk factors turn into causes of stroke onset has not been well studied. Gene expression patterns may be the key to this question. On the other hand, it has been widely accepted that a prior history of stroke or transient ischemic attack (TIA) increases the risk of secondary stroke even when there is no observable sign of lasting damage. An investigation on the molecular-scale alternations following stroke or TIA would be a necessary step for revealing the underlying cause and exploring better therapeutic solutions. Despite the surging demands and the rapidly declining cost of gene expression profiling, molecular studies on human subjects are still very limited due to both technical and ethical difficulties. Animal models, under sophisticatedly designed experiment conditions, are still not a long-term substitutable platform for stroke genomics studies. An efficient method for translating the findings of animal experiments to human studies is an essential demand because human subjects are still rare, even if only used for validation purposes.

In this paper, a method was proposed of discovering cross-platform biomarkers by taking advantage of gene interactions that are relatively stable across species and tissues. The miRNAs can perform their regulatory roles on two molecular levels: the mRNA and protein levels. However, due to the limitations of current datasets, only the mRNA level was considered in this paper. As is demonstrated in the experiment results, the proposed approach not only generates more replicable biomarkers across species and tissues, but it also captures key genes that are actively involved in various biological signal pathways, which is more helpful for in-depth studies of post-stroke pathology. By applying the method to peripheral blood samples collected from human stroke patients of different stages, it was discovered that molecular signals representing stroke-induced damages and responses, which originate from cerebral tissues, propagate into peripheral blood, and the effects persist into the recovery stages. This not only justifies the possibility of an efficient early diagnosis and prognosis of ischemic stroke using peripheral blood sampling, but it also provides some clue for explaining the cause of elevated risk for stroke and TIA patients after recovery, suggesting the need of exploring molecular therapeutic targets that help with rebuilding the gene regulatory patterns and inhibiting detrimental molecular signals.

The fact that biomarkers derived from animal models can accurately indicate the stroke status of human subjects further validates the important value of molecular-level animal experiments in stroke studies. Currently, most animal studies are carried out with manipulated acute ischemic injuries, which may have some distinctions from human stroke in the real world. With future developments in experimental technologies, researchers would be able to emulate chronic stroke risk factors to obtain a more comprehensive knowledge of stroke pathology and prevention. In that situation, the present approach will be more advantageous in providing an efficient tool for exploring predictive biomarkers as well as biological pathways that can be easily translated to human patients, while also reducing the cost and time of the entire validation cycle.

## Additional Information

**How to cite this article**: Wang, Y. and Cai, Y. Obtaining Human Ischemic Stroke Gene Expression Biomarkers from Animal Models: A Cross-species Validation Study. *Sci. Rep.* **6**, 29693; doi: 10.1038/srep29693 (2016).

## Supplementary Material

Supplementary Information

## Figures and Tables

**Table 1:** Top ranked mRNAs selected by the PageRank scores.

Top mRNAs	Test set 1		Test set 2	
	p-value	Dys-regulate in stroke samples	p-value	Dys-regulate in stroke samples
MAFK	8.97E-18	Up-regulated	7.79E-07	Up-regulated
TESC	4.09E-36	Down-regulated	3.02E-08	Down-regulated
SIK1	1.68E-15	Down-regulated	9.61E-05	Down-regulated
PER1	2.09E-17	Up-regulated	5.36E-06	Up-regulated
NUMB	4.76E-08	Up-regulated	0.702168172	Up-regulated
DMP1	2.51E-52	Up-regulated	4.66E-08	Up-regulated
JUN	2.80E-29	Down-regulated	0.000669585	Down-regulated
LIPE	7.75E-09	Up-regulated	1.03E-06	Up-regulated
PLAT	7.80E-39	Down-regulated	1.90E-08	Down-regulated
RTEL1	0.36504457	Up-regulated	0.8546838	Up-regulated
WDR91	6.70E-07	Up-regulated	0.007632295	Up-regulated
BTG2	1.70E-24	Down-regulated	0.000212577	Down-regulated
IQSEC3	4.52E-50	Up-regulated	1.09E-09	Up-regulated
NPAS4	3.98E-38	Down-regulated	1.15E-08	Down-regulated
CAMKK1	1.46E-20	Down-regulated	1.55E-06	Down-regulated
TTC22	1.62E-19	Down-regulated	3.46E-08	Down-regulated
ADRA1B	2.14E-34	Down-regulated	2.13E-08	Down-regulated
TCF25	0.001314673	Up-regulated	0.983744378	Up-regulated
CRHBP	6.78E-29	Up-regulated	1.13E-08	Up-regulated
SMOX	0.131954977	Up-regulated	0.386940958	Up-regulated

**Table 2:** Biological classification of selected features.

Classification	Biomarker type	mRNAs/miRNA (Rank) (Validated Species tissue)
Biomarkers transcribed from stroke related genomic mutations	Gene	PCSK2 (44) (human blood), LIMK1 (61) (human blood)
Biomarkers involved in processes causing stroke onset and development	Gene	GADD45B (53) (rat brain), CYP46A1 (100) (rat/mouse brain)
	miRNA	miR-494-3p (18) (human blood)
Biomarkers involved in biological processes accompanied with or after stroke	Gene	LIPE (8) (human blood), CAMK1G (32) (mouse brain), ASPA (33) (-), NOTCH4 (36) (mouse brain/blood), PLA1A (38) (human blood), TYRO3 (49) (human blood), CORO6 (71) (-), SIK1 (3) (-), SCG2 (24) (human/rat brain), CIRBP (27) (mouse brain), PGLYRP1 (30) (human blood), ARTN (34) (rat brain), COQ7 (62) (mouse brain), BAI1 (64) (-), TSPAN2 (69) (rat brain)
	miRNA	miR-129-5p (14) (human blood), miR-29a-5p (4) (-), miR-138-5p (36) (-)
Biomarkers involved in stroke recovery	Gene	NUMB (5) (human blood), GNE (23) (human cerebrospinal fluid), CAMK2G (48) (rat brain)
Potential stroke therapeutic targets	Gene	SIK1 (3) (-), BAI1 (64) (-), PLAT (9) (human blood), ADRA1B (17) (rat brain)
Biomarkers that have been previously reported to be differentially expressed among stroke and healthy subjects/across stroke samples and subtypes	Gene	PER1 (4) (-), BTG2 (12) (rat brain) NPAS4 (14) (rat brain), CRHBP (19) (rat brain), SMOX (20) (human blood), DUSP1 (75) (human blood), CRY1 (92) (human carotid plaques)
	miRNA	miR-665 (1) (human blood), miR-21-5p (2) (human blood), miR-184 (5) (human blood), miR-877-5p (7) (human blood), miR-300-5p (9) (human blood), miR-130b-3p (11) (human blood), miR-223-3p (12) (human blood, mouse brain), miR-129-5p (14) (human blood), miR-494-3p (18) (human blood), miR-326 (20) (human blood), miR-30c-1-3p (21) (human blood), miR-551b-3p (23) (human blood), miR-200b-3p (24) (human blood), miR-124-3p (26) (human blood), let-7b-5p (30) (human blood), let-7i-5p (33) (human blood), miR-125b-5p (34) (human blood, rat brain), let-7a-5p (35) (human blood), miR-134-5p (37) (mouse brain), miR-103a-3p (40) (human blood), miR-107 (41) (human blood), miR-106b-3p (43) (human blood), miR-125a-3p (44) (Human umbilical cord vessels), miR-144-3p (45) (human blood), miR-1224-5p (49) (rat brain)
Biomarkers in the same family of known stroke-related markers	Gene	CAMKK1 (15) (mouse brain), TTC22 (16) (human blood), TOB2 (22) (human brain), GADD45G (25) (rat brain), PDE4B (26) (mouse brain), ANXA11 (28) (mouse/rat brain, human blood)
	miRNA	miR-675-5p (3) (mouse-brain), miR-290-5p (6) (rat-brain), miR-483-3p (22) (human blood)
Biomarkers interacted with/binding with/regulate stroke-related factors	Gene	TESC (2) (-), NUMB (5) (human blood), JUN (7) (-), GNE (23) (human cerebrospinal fluid), GNL3 (29) (mouse brain), AZIN1 (31) (human brain), NFIL3 (35) (-), BHLHE40 (37) (-), CMIP (41) (mouse brain), MRPL41 (42) (rat brain)

**Table 3:** Core gene groups of selected features.

Core group	Functional terms	Genes
Protein kinase related genes	kinase/kinase activity	KCNH1, CAMK2G, CAMK1G, CAMKK1, MAP3K6, MAPK8IP1, SIK1, MARK1, LIMK1, TYRO3, GNE, PFKP
	phosphorus/-ate metabolic process	CAMK2G, CAMK1G, CAMKK1, MAP3K6, MAPK8IP1, SIK1, MARK1, LIMK1, TYRO3
	calmodulin-binding	KCNH1, CAMK2G, CAMK1G, CAMKK1
Genes associated with cell cycles	developmental/differentiation	NOTCH4, GADD45G, GADD45B, RTN4RL2, JUN, CREM
	Apoptosis/cell death	GADD45G, GADD45B, RTEL1, JUN
	regulation of cell proliferation	NOTCH4, SCG2, SESN1, BTG2, JUN
	blood vessel morphogenesis/development	ADRA1B, NOTCH4, SCG2, JUN
	response to stress/abiotic stimulus	ADRA1B, GADD45G, DUSP1, CIRBP, SESN1, BTG2, RTEL1, RTN4RL2, JUN
	intracellular signaling	ADRA1B, NOTCH4, SCG2, GADD45G, GADD45B, DUSP1, JUN, CREM
Circadian rhythms genes	biological/circadian rhythms	JUN, CREM, NFIL3, CRY1, PER1, BHLHE40, HS3ST2, CCRN4L, PGLYRP1
