# Supplementary material for: Obtaining Human Ischemic Stroke Gene Expression Biomarkers from Animal Models: A Cross-species Validation Study
Source: Sci Rep. 2016 Jul 13;6:29693. doi: 10.1038/srep29693 (PMC4942769; doi:10.1038/srep29693)
Supplement: Supplementary Information [file srep29693-s1.pdf]

Obtaining Human Ischemic Stroke Gene Expression Biomarkers from Animal Models: A Cross-species Validation Study

Yingying Wang, Yunpeng Cai

Table S1 The detail information of top-ranked mRNAs

**Rank**	**Gene Symbol**	**Entrez Gene ID**	**Category**	**PMID/URLs**	**Species**	**Tissue**	**Experiment Type**	**Number of related top miRNAs**
1	MAFK	7975	-	-	-	-	-	16
2	TESC	54997	Genes interacted with /binding with/ regulate stroke-related factors	PMID:17339567; 15674430	-	-	-	7
3	SIK1	150094	①Genes involved in biological processes accompanied with or after stroke<br>②Potential stroke therapeutic targets	http://connection.ebscohost.com/c/articles/58018898/pass-up-salt-inducible-kinase-stroke-survival; http://link.springer.com/chapter/10.1007%2F978-1-4419-9663-3_15#page-1	-	-	-	16
4	PER1	5187	Genes that have been previously reported to be differentially expressed among stroke and healthy subjects	PMID: 17936274	-	-	-	16
5	NUMB	8650	Genes involved in stroke recovery; Genes interacted with /binding with/ regulate stroke-related factors	PMID: 19138666; 26051664	Human	Blood	Immunoassay	22

6	DMP1	1758	-	-	-	-	-	9
7	JUN	3725	Genes interacted with /binding with/ regulate stroke-related factors	PMID: 12944025	-	-	-	7
8	LIPE	3991	Genes involved in biological processes accompanied with or after stroke	PMID: 26274054	Human	Blood	-	7
9	PLAT	5327	Potential stroke therapeutic targets	PMID: 26247772; 15016487	Human	Blood	-	8
10	RTEL1	51750	-	-	-	-	-	0
11	WDR91	29062	-	-	-	-	-	17
12	BTG2	7832	Genes that have been previously reported to be differentially expressed among stroke and healthy subjects	PMID: 24112606	Rat	Brain	Western blotting	17
13	IQSEC3	440073	-	-	-	-	-	12
14	NPAS4	266743	Genes that have been previously reported to be differentially expressed among stroke and healthy subjects	PMID: 17156197; 24669275; 23431968	Rat	Brain	Expression profiles; Northern blots; PCR; Immunohistochemistry; RT-PCR	17
15	CAMKK1	84254	Genes encoding proteins that were in the same family of known stroke-related proteins	PMID: 25331941	Mouse	Brain	Hemoglobin assay; Immunoblotting	11
16	TTC22	55001	Genes encoding proteins that were in the same family of known stroke-related proteins	PMID: 22453632	Human	Blood	Microarray	9
17	ADRA1B	147	Potential stroke therapeutic targets	PMID: 22860001	Rat	Brain	-	6
18	TCF25	22980	-	-	-	-	-	7

19	CRHBP	1393	Genes that have been previously reported to be differentially expressed among stroke and healthy subjects	PMID: 23251410	Rat	Brain	Microarray	3
20	SMOX	54498	Genes that have been previously reported to be differentially expressed among stroke and healthy subjects	PMID: 16269634	Human	Blood	Liquid chromatography and ELISA	11
21	CHPF2	54480	-	-	-	-	-	6
22	TOB2	10766	Genes encoding proteins that were in the same family of known stroke-related proteins	PMID: 19468252	Human	Brain	Immunohistochemistry; Western blotting	19
23	GNE	10020	①Genes involved in stroke recovery ②Genes interacted with /binding with/ regulate stroke-related factors	PMID: 22224242	Human	Cerebro spinal fluid	-	22
24	SCG2	7857	Genes involved in biological processes accompanied with or after stroke	PMID: 18079966	Human; Rat	Brain	Western blot	11
25	GADD45G	10912	Genes encoding proteins that were in the same family of known stroke-related proteins	PMID: 25678854	Rat	Brain	Immunohistochemistry; Western blot	4
26	PDE4B	5142	Genes encoding proteins that were in the same family of known stroke-related proteins	PMID: 24011921	Mouse	Brain	-	22
27	CIRBP	1153	Genes involved in biological processes accompanied with or after stroke	PMID: 24613680	Mouse	Brain	Immunohistochemistry; PCR; Western blot	22
28	ANXA11	311	Genes encoding proteins that were in the same family of known stroke-related	PMID: 26205642; 24368559; 25345638	Mouse; Rat;	Brain; Blood	RT-PCR; Western blot,	17

			proteins		Human		Immunofluorescence; Immunohistochemistry; PCR; enzyme-linked immunosorbent assay	
29	GNL3	26354	Genes interacted with /binding with/ regulate stroke-related factors	PMID: 25779964	Mouse	Brain	Immunohistochemical	1
30	PGLYRP1	8993	Genes involved in biological processes accompanied with or after stroke	PMID: 22167233	Human	Blood	Microarray	3
31	AZIN1	51582	Genes interacted with /binding with/ regulate stroke-related factors	PMID: 19832840	Human	Brain	Immunohistochemistry; Western blotting; RT-PCR	19
32	CAMK1G	57172	Genes involved in biological processes accompanied with or after stroke	PMID: 23868268	Mouse	Brain	-	7
33	ASPA	443	Genes involved in biological processes accompanied with or after stroke	PMID: 25497124	-	-	-	4
34	ARTN	9048	Genes involved in biological processes accompanied with or after stroke	PMID: 11564414	Rat	Brain	Immunohistochemistry	6
35	NFIL3	4783	Genes interacted with /binding with/ regulate stroke-related factors	PMID: 17936274	-	-	-	6
36	NOTCH4	4855	Genes involved in biological processes accompanied with or after stroke	PMID: 25468970	Mouse	Brain; Blood	Immunostaining	29
37	BHLHE40	8553	Genes interacted with /binding with/	PMID: 17936274	-	-	-	15

			regulate stroke-related factors					
38	PLA1A	51365	Genes involved in biological processes accompanied with or after stroke	PMID: 26274054	Human	Blood	-	10
39	MRPS34	65993		-	-	-	-	4
40	RIMBP2	23504		-	-	-	-	23
41	CMIP	80790	Genes interacted with /binding with/ regulate stroke-related factors	PMID: 24319262	Mouse	Brain	Quantitative PCR; Western Blot	13
42	MRPL41	64975	Genes interacted with /binding with/ regulate stroke-related factors	PMID: 15547950; 25722793	Rat	Brain	Immunohistochemistry; Western Blot; RT-PCR	0
43	CNKSR3	154043	-	-	-	-	-	14
44	PCSK2	5126	Genes transcribed from stroke related genomic mutations	PMID: 20036365	Human	Blood	Microarray	15
45	KIFC2	90990	-	-	-	-	-	10
46	RASL11A	387496	-	-	-	-	-	1
47	FEM1B	10116	-	-	-	-	-	1
48	CAMK2G	818	Genes involved in stroke recovery	PMID: 25423262	Rat	Brain	Bead arrays; PCR	20
49	TYRO3	7301	Genes involved in biological processes accompanied with or after stroke	PMID: 20664904	Human	Blood	RT-PCR	19
50	SEC61A2	55176	-	-	-	-	-	23
51	SORBS2	8470	-	-	-	-	-	23
52	LENG1	79165	-	-	-	-	-	1
53	GADD45B	4616	Genes involved in processes causing stroke onset and development	PMID: 25678854	Rat	Brain	Immunohistochemistry; Western	7

							blot	
54	INPP4A	3631	-	-	-	-	-	8
55	PMEPA1	56937	-	-	-	-	-	15
56	SYT12	91683	-	-	-	-	-	11
57	ZMYND8	23613	-	-	-	-	-	21
58	KCNH1	3756	-	-	-	-	-	8
59	CADM4	199731	-	-	-	-	-	11
60	BEND5	79656	-	-	-	-	-	6
61	LIMK1	3984	①Genes transcribed from stroke related genomic mutations<br>②Genes involved in biological processes accompanied with or after stroke	PMID: 18566305	Human	Blood	Microarray	16
62	COQ7	10229	-	PMID: 20170652	Mouse	Brain	-	12
63	HS3ST2	9956	-	-	-	-	-	15
64	BAI1	575	①Genes involved in biological processes accompanied with or after stroke<br>②Potential stroke therapeutic targets	PMID: 21509575	-	-	-	0
65	UPP1	7378	-	-	-	-	-	3
66	PITPNM1	9600	-	-	-	-	-	6
67	TLE3	7090	-	-	-	-	-	15
68	MAP3K6	9064	-	-	-	-	-	10
69	TSPAN2	10100	Genes involved in biological processes accompanied with or after stroke	PMID: 20883670	Rat	Brain	Microarray; qRT-PCR	16
70	FXR2	9513	-	-	-	-	-	23

71	CORO6	84940	Genes involved in biological processes accompanied with or after stroke	PMID: 25882105; 24886966	-	-	-	11
72	PLEKHG3	26030	-	-	-	-	-	11
73	CREM	1390	-	-	-	-	-	22
74	MARK1	4139	-	-	-	-	-	25
75	DUSP1	1843	Genes that have been previously reported to be differentially expressed among stroke and healthy subjects	PMID: 25135788	Human	Blood	Microarray; (RT)-qPCR	12
76	RASSF5	83593	-	-	-	-	-	0
77	PAK1IP1	55003	-	-	-	-	-	2
78	MARCKS L1	65108	-	-	-	-	-	20
79	GORAB	92344	-	-	-	-	-	16
80	DSCAM	1826	-	-	-	-	-	14
81	WASF1	8936	-	-	-	-	-	11
82	RTN4RL2	349667	-	-	-	-	-	2
83	STRN3	29966	-	-	-	-	-	17
84	COQ10B	80219	-	-	-	-	-	6
85	ALDH1A1	216	-	-	-	-	-	6
86	PHRF1	57661	-	-	-	-	-	5
87	KCNK12	56660	-	-	-	-	-	7
88	GRIK5	2901	-	-	-	-	-	10
89	LURAP1L	286343	-	-	-	-	-	16
90	MAPK8IP1	9479	-	-	-	-	-	3
91	KLF9	687	-	-	-	-	-	18

92	CRY1	1407	Genes that have been previously reported to be differentially expressed among stroke and healthy subjects	PMID: 24418196	Human	Carotid plaques	Immunofluorescence; qRT-PCR	9
93	GPR162	27239	-	-	-	-	-	1
94	SESN1	27244	-	-	-	-	-	22
95	SLC35F4	341880	-	-	-	-	-	5
96	CCRN4L	25819	-	-	-	-	-	0
97	TENM3	55714	-	-	-	-	-	16
98	ADRM1	11047	-	-	-	-	-	9
99	PFKP	5214	-	-	-	-	-	16
100	CYP46A1	10858	Genes involved in processes causing stroke onset and development	PMID: 22343944	Rat; Mouse	Brain	-	21

Table S2 The detail information of top-ranked miRNAs

**Rank**	**miRNA_id**	**Previous ID**	**Sequence**	**Family**	**Category**	**PMID**	**Number of targets in top 100 genes**
1	mmu-miR-665-3p	mmu-miR-665	accaggaggcugaggucccu	mir-665	miRNAs that have been previously reported to be differentially expressed across stroke samples and subtypes	19888324	33
2	mmu-miR-21-5p	mmu-miR-21*	uagcuuaucagacugauguuga	mir-21	miRNAs that have been previously reported to be differentially expressed across stroke samples and subtypes	19888324; 23860376	12

3	mmu-miR-675-5p	-	uggugcggaaagggcccacagu	mir-675	miRNAs in the same family of known stroke markers	24574964	33
4	mmu-miR-291a-5p	-	caucaaaguggaggcccucucu	mir-290	miRNAs involved in biological processes accompanied with or after stroke	24373752	29
5	hsa-miR-184	-	uggacggagaacugauaagggu	mir-184	miRNAs that have been previously reported to be differentially expressed across stroke samples and subtypes	19888324	28
6	mmu-miR-290a-5p	mmu-miR-290-5p	acucaaacuaugggggcacuuu	mir-290	miRNAs in the same family of known stroke markers	18258830	0
7	hsa-miR-877-5p	hsa-miR-877	guagaggagauggcgcaggg	mir-877	miRNAs that have been previously reported to be differentially expressed across stroke samples and subtypes	19888324	30
8	mmu-miR-294-5p	mmu-miR-294*	acucaaaauggaggcccuaucu	mir-290	miRNAs in the same family of known stroke markers	18258830	0
9	mmu-miR-300-5p	mmu-miR-300*	uugaagagagguuauccuuugu	mir-154	miRNAs that have been previously reported to be differentially expressed across stroke samples and subtypes	19888324	0
10	rno-miR-204-5p	rno-miR-204*	uucccuuugucauccuaugccu	mir-204	--	--	0
11	hsa-miR-130b-3p	hsa-miR-130b	cagugcaaugaugaaagggcau	mir-130	miRNAs that have been previously reported to be differentially expressed across stroke samples and subtypes	19888324	33

12	hsa-miR-223-3p	hsa-miR-223	ugucaguuugucaaauacccca	mir-223	miRNAs that have been previously reported to be differentially expressed across stroke samples and subtypes	24708646	27
13	mmu-miR-503-5p	mmu-miR-503	uagcagcgggaacaguacugcag	mir-503	--	--	40
14	hsa-miR-129-5p	-	cuuuuugcggucugggcuugc	mir-129	miRNAs involved in biological processes accompanied with or after stroke	19523330	38
15	mmu-miR-351-5p	mmu-miR-351	ucccugaggagcccuuugagccug	mir-351	--	--	0
16	hsa-miR-542-5p	-	ucggggaucaucaugucacgaga	mir-542	--	--	20
17	rno-miR-466c-5p	rno-miR-466c	ugugauguguguauguacaug	mir-467	--	--	0
18	rno-miR-494-3p	rno-miR-494	ugaaacauacacgggaaaccucu	mir-154	miRNAs involved in processes causing stroke onset and development	26583674	32
19	hsa-miR-299-5p	-	ugguuuaccgucccacauacau	mir-299	--	--	21
20	hsa-miR-326	-	ccucugggcccuuccuccag	mir-326	miRNAs that have been previously reported to be differentially expressed across stroke samples and subtypes	19888324	51
21	hsa-miR-30c-1-3p	hsa-miR-30c-1*	cugggagaggguuguuuacucc	mir-30	miRNAs that have been previously reported to be differentially expressed across stroke samples and subtypes	19888324	17
22	mmu-miR-483-3p	mmu-miR-483*	ucacuccuccccucccgucuu	mir-483	miRNAs in the same family of known stroke markers	26096228	25

23	hsa-miR-551b-3p	hsa-miR-551b	gcgacccauacuugguuucag	mir-551	miRNAs that have been previously reported to be differentially expressed across stroke samples and subtypes	19888324	13
24	hsa-miR-200b-3p	hsa-miR-200b	uaauacugccugguaaugauga	mir-8	miRNAs that have been previously reported to be differentially expressed across stroke samples and subtypes	25697638	32
25	hsa-miR-874-3p	hsa-miR-874	cugcccuggcccgagggaccga	mir-874	--	--	28
26	hsa-miR-124-3p	hsa-miR-124	uaaggcacgcggugaaugcc	mir-124	miRNAs that have been previously reported to be differentially expressed across stroke samples and subtypes	26968104	37
27	rno-miR-742-3p	rno-miR-742	gaaagccaccauguugggguaaa	mir-742	--	--	0
28	mmu-miR-325-5p	mmu-miR-325	ccuaguaggugcucaguaagugu	mir-325	--	--	12
29	mmu-miR-329-3p	mmu-miR-329	aacacacccagcuaaccuuuuu	mir-329	--	--	42
30	hsa-let-7b-5p	hsa-let-7b	ugagguaguagguugugugguu	let-7	miRNAs that have been previously reported to be differentially expressed across stroke samples and subtypes	19888324	35
31	hsa-miR-22-3p	hsa-miR-22	aagcugccaguugaagaacugu	mir-22	miRNAs involved in biological processes accompanied with or after stroke	26191152	39
32	hsa-miR-128-3p	hsa-miR-128	ucacagugaaccggucucuuu	mir-128	--	--	30
33	hsa-let-7i-5p	hsa-let-7i	ugagguaguaguuugugcuguu	let-7	miRNAs that have been previously reported to be	19888324	30

					differentially expressed across stroke samples and subtypes		
34	hsa-miR-125b-5p	hsa-miR-125b	ucccugagacccuaacuuguga	mir-10	miRNAs that have been previously reported to be differentially expressed across stroke samples and subtypes	24447930	30
35	hsa-let-7a-5p	hsa-let-7a	ugagguaguagguuguauaguu	let-7	miRNAs that have been previously reported to be differentially expressed across stroke samples and subtypes	19888324	28
36	hsa-miR-138-5p	hsa-miR-138	agcugguguugugaaucaggccg	mir-138	miRNAs involved in biological processes accompanied with or after stroke	22218763	25
37	hsa-miR-134-5p	hsa-miR-134	ugugacugguugaccagagggg	mir-134	miRNAs that have been previously reported to be differentially expressed across stroke samples and subtypes	25003713	32
38	mmu-miR-322-5p	mmu-miR-322	cagcagcaauucauguuuugga	mir-322	--	--	0
39	mmu-miR-664-3p	mmu-miR-664	uauucauuuacuccccagccua	mir-664	--	--	27
40	hsa-miR-103a-3p	hsa-miR-103	agcagcauuguacagggcuauga	mir-103	miRNAs that have been previously reported to be differentially expressed across stroke samples and subtypes	19888324	40
41	hsa-miR-107	-	agcagcauuguacagggcuauca	mir-103	miRNAs that have been previously reported to be differentially expressed across	19888324	40

					stroke samples and subtypes		
42	hsa-miR-28-5p	-	aaggagcucacagucuauugag	mir-28	--	--	15
43	hsa-miR-106b-3p	hsa-miR-106b*	ccgcacuguggguacuugcugc	mir-17	miRNAs that have been previously reported to be differentially expressed across stroke samples and subtypes	19888324	7
44	hsa-miR-125a-3p	-	acaggugagguucuugggagcc	mir-10	miRNAs that have been previously reported to be differentially expressed across stroke samples and subtypes	27106952	37
45	hsa-miR-144-3p	hsa-miR-144	uacaguauagaugauguacu	mir-144	miRNAs that have been previously reported to be differentially expressed across stroke samples and subtypes	19888324	33
46	mmu-miR-672-5p	mmu-miR-672	ugagguugguguacugugugug a	mir-672	--	--	12
47	rno-miR-760-5p	-	ccccucaggccaccagagcccg	mir-760	--	--	0
48	hsa-miR-760	-	cggcucugggucugugggga	mir-760	--	--	31
49	mmu-miR-1224-5p	mmu-miR-1224	gugaggacuggggagguggag	mir-122 4	miRNAs that have been previously reported to be differentially expressed across stroke samples and subtypes	22937209	20

Table S3 Function chart of top 100 mRNAs

Category	Term	Count	P-Value	Genes Symbol	Benjamini
GOTERM_BP_FAT	GO:0048511~rhythmic process	7	0.0001	JUN, HS3ST2, CCRN4L, CRY1, PGLYRP1, PER1, NFIL3	0.0704
UP_TISSUE	Brain	60	0.0001	PITPNM1, CYP46A1, FXR2, PCSK2, IQSEC3, TYRO3, CREM, ZMYND8, GADD45B, GPR162, ARTN, CNKSR3, KIFC2, PLAT, DSCAM, TENM3, WDR91, INPP4A, PLEKHG3, HS3ST2, MAFK, TCF25, RIMBP2, NPAS4, MAP3K6, PLA1A, TOB2, GADD45G, PHRF1, ASPA, COQ10B, RASL11A, PFKP, ADRM1, MARCKSL1, PDE4B, COQ7, RTEL1, ALDH1A1, NUMB, PER1, ANXA11, RASSF5, RTN4RL2, SYT12, DUSP1, LIMK1, KCNH1, MAPK8IP1, FEM1B, AZIN1, SCG2, ADRA1B, CRY1, MARK1, CAMK2G, CAMKK1, CMIP, TLE3, SORBS2	0.0091
SP_PIR_KEYWORDS	Biological rhythms	4	0.0001	CCRN4L, CRY1, PER1, NFIL3	0.0247
GOTERM_BP_FAT	GO:0007623~circadian rhythm	5	0.0001	JUN, HS3ST2, CRY1, PGLYRP1, PER1	0.0515
UP_SEQ_FEATURE	Region of interest: Calmodulin-binding	5	0.0002	STRN3, CAMK1G, CAMK2G, KCNH1, CAMKK1	0.0753
GOTERM_BP_FAT	GO:0042752~regulation of circadian rhythm	4	0.0002	BHLHE40, PGLYRP1, PER1, CREM	0.0554
SP_PIR_KEYWORDS	Calmodulin-binding	6	0.0004	STRN3, CAMK1G, CAMK2G, KCNH1, CAMKK1, MARCKSL1	0.0339
SP_PIR_KEYWORDS	Kinase	12	0.0008	ZMYND8, LIMK1, CAMK1G, MARK1, CAMK2G, SIK1, GNE, CAMKK1, MAPK8IP1, TYRO3, PFKP, MAP3K6	0.0472
GOTERM_MF_FAT	GO:0005516~calmodulin binding	6	0.0011	STRN3, CAMK1G, CAMK2G, KCNH1, CAMKK1, MARCKSL1	0.2543

GOTERM_BP_FAT	GO:0007243~protein kinase cascade	9	0.0014	GADD45B, NOTCH4, SCG2, ADRA1B, GADD45G, MARK1, SIK1, MAPK8IP1, MAP3K6	0.2232
UP_SEQ_FEATURE	DNA-binding region: Basic motif	6	0.0015	JUN, BHLHE40, MAFK, NFIL3, NPAS4, CREM	0.2688
GOTERM_BP_FAT	GO:0006468~protein amino acid phosphorylation	12	0.0016	GADD45B, LIMK1, CAMK1G, GADD45G, MARK1, CAMK2G, SIK1, CAMKK1, MAPK8IP1, LIPE, TYRO3, MAP3K6	0.2111
SP_PIR_KEYWORDS	Phosphoprotein	53	0.0019	JUN, PITPNM1, FXR2, NOTCH4, CAMK1G, IQSEC3, TYRO3, CREM, ZMYND8, CNKSR3, BAI1, INPP4A, WDR91, PLEKHG3, MAFK, TCF25, RIMBP2, MAP3K6, DMP1, TOB2, PHRF1, GNL3, PFKP, ADRM1, MARCKSL1, LENG1, CADM4, PDE4B, BHLHE40, NUMB, PER1, LIPE, ANXA11, RASSF5, LIMK1, NFIL3, LURAP1L, MAPK8IP1, PAK1IP1, SCG2, STRN3, ADRA1B, CRY1, MARK1, CAMK2G, GNE, SESN1, SIK1, CAMKK1, CMIP, TLE3, SORBS2, KCNK12	0.0815
INTERPRO	IPR004827:Basic-leucine zipper (bZIP) transcription factor	4	0.0034	JUN, MAFK, NFIL3, CREM	0.6080
KEGG_PATHWAY	hsa04710: Circadian rhythm	3	0.0038	BHLHE40, CRY1, PER1	0.1930
UP_SEQ_FEATURE	Nucleotide phosphate-binding region: ATP	13	0.0040	CAMK1G, RTEL1, MARK1, CAMK2G, GNE, TYRO3, MAP3K6, LIMK1, CNKSR3, KIFC2, SIK1, CAMKK1, PFKP	0.4293
SMART	SM00338: BRLZ	4	0.0044	JUN, MAFK, NFIL3, CREM	0.2493
SP_PIR_KEYWORDS	Cytoplasm	29	0.0045	PITPNM1, FXR2, CAMK1G, ALDH1A1, GORAB, PER1, IQSEC3, LIPE, ANXA11, RASSF5, LIMK1, SMOX, CIRBP, MAPK8IP1,	0.1487

				FEM1B, TESC, STRN3, CRY1, MARK1, GNE, WASF1, DMP1, TOB2, ASPA, SIK1, CAMKK1, CMIP, ADRM1, SORBS2	
GOTERM_MF_FAT	GO:0004683~calmodulin-dependent protein kinase activity	3	0.0055	CAMK1G, CAMK2G, CAMKK1	0.5136
GOTERM_BP_FAT	GO:0007242~intracellular signaling cascade	16	0.0056	KLF9, PMEPA1, NOTCH4, SCG2, ADRA1B, MARK1, MAP3K6, RASSF5, CREM, DUSP1, GADD45B, LIMK1, GADD45G, RASL11A, SIK1, MAPK8IP1	0.4898
GOTERM_BP_FAT	GO:0016310~phosphorylation	12	0.0066	GADD45B, LIMK1, CAMK1G, GADD45G, MARK1, CAMK2G, SIK1, CAMKK1, MAPK8IP1, LIPE, TYRO3, MAP3K6	0.4937
UP_SEQ_FEATURE	Domain: PAC	3	0.0080	KCNH1, PER1, NPAS4	0.5636
GOTERM_BP_FAT	GO:0006793~phosphorus metabolic process	13	0.0105	DUSP1, GADD45B, LIMK1, CAMK1G, GADD45G, MARK1, CAMK2G, SIK1, CAMKK1, MAPK8IP1, LIPE, TYRO3, MAP3K6	0.6137
GOTERM_BP_FAT	GO:0006796~phosphate metabolic process	13	0.0105	DUSP1, GADD45B, LIMK1, CAMK1G, GADD45G, MARK1, CAMK2G, SIK1, CAMKK1, MAPK8IP1, LIPE, TYRO3, MAP3K6	0.6137
UP_SEQ_FEATURE	Domain: Protein kinase	8	0.0110	LIMK1, CAMK1G, MARK1, CAMK2G, SIK1, CAMKK1, TYRO3, MAP3K6	0.6009
KEGG_PATHWAY	hsa04115: p53 signaling pathway	4	0.0128	GADD45B, GADD45G, BAI1, SESN1	0.3038
UP_SEQ_FEATURE	Compositionally biased region: Poly-Arg	5	0.0130	GRIK5, FXR2, NOTCH4, CNKSR3, ADRA1B	0.5964
SP_PIR_KEYWORDS	Serine/threonine-protein kinase	7	0.0139	LIMK1, CAMK1G, MARK1, CAMK2G, SIK1, CAMKK1, MAP3K6	0.3387
INTERPRO	IPR000014: PAS	3	0.0151	KCNH1, PER1, NPAS4	0.8755
INTERPRO	IPR017441: Protein kinase, ATP binding site	8	0.0152	LIMK1, CAMK1G, MARK1, CAMK2G, SIK1, CAMKK1, TYRO3, MAP3K6	0.7530

INTERPRO	IPR008271: Serine/ threonine protein kinase, active site	7	0.0158	LIMK1, CAMK1G, MARK1, CAMK2G, SIK1, CAMKK1, MAP3K6	0.6622
INTERPRO	IPR002290: Serine/ threonine protein kinase	6	0.0163	CAMK1G, MARK1, CAMK2G, SIK1, CAMKK1, MAP3K6	0.5926
INTERPRO	IPR017442: Serine/ threonine protein kinase-related	7	0.0168	LIMK1, CAMK1G, MARK1, CAMK2G, SIK1, CAMKK1, MAP3K6	0.5372
SP_PIR_KEYWORDS	Allosteric enzyme	3	0.0172	CAMK1G, GNE, PFKP	0.3546
GOTERM_BP_FAT	GO:0033554~cellular response to stress	9	0.0172	JUN, BTG2, GADD45G, RTEL1, CRY1, SESN1, MAPK8IP1, MAP3K6, RTN4RL2	0.7527
PIR_SUPERFAMILY	PIRSF010369: growth arrest and DNA-damage-inducible protein GADD45	2	0.0177	GADD45B, GADD45G	0.6704
SMART	SM00091: PAS	3	0.0181	KCNH1, PER1, NPAS4	0.4468
GOTERM_BP_FAT	GO:0008285~negative regulation of cell proliferation	7	0.0188	TESC, JUN, TOB2, SCG2, BTG2, BAI1, SESN1	0.7474
INTERPRO	IPR000719: Protein kinase, core	8	0.0190	LIMK1, CAMK1G, MARK1, CAMK2G, SIK1, CAMKK1, TYRO3, MAP3K6	0.5276
UP_SEQ_FEATURE	Domain: Leucine-zipper	4	0.0195	JUN, MAFK, NFIL3, CREM	0.6892
GOTERM_MF_FAT	GO:0004672~protein kinase activity	9	0.0201	LIMK1, CAMK1G, MARK1, CAMK2G, KCNH1, SIK1, CAMKK1, TYRO3, MAP3K6	0.8263
UP_SEQ_FEATURE	Active site: Proton acceptor	9	0.0209	CAMK1G, ALDH1A1, MARK1, CAMK2G, SIK1, CAMKK1, TYRO3, PFKP, MAP3K6	0.6655

UP_SEQ_FEATURE	Binding site: ATP	8	0.0225	LIMK1, CAMK1G, MARK1, CAMK2G, SIK1, CAMKK1, TYRO3, MAP3K6	0.6503
GOTERM_BP_FAT	GO:0000165~MAPKKK cascade	5	0.0226	GADD45B, SCG2, GADD45G, MAPK8IP1, MAP3K6	0.7777
SMART	SM00220:S_TKc	6	0.0228	CAMK1G, MARK1, CAMK2G, SIK1, CAMKK1, MAP3K6	0.3932
SP_PIR_KEYWORDS	Repressor	7	0.0249	CRY1, MAFK, CIRBP, TCF25, NFIL3, TLE3, CREM	0.4276
SP_PIR_KEYWORDS	Nucleotide-binding	16	0.0254	CAMK1G, RTEL1, CRY1, MARK1, CAMK2G, GNE, TYRO3, MAP3K6, LIMK1, CNKSR3, KIFC2, RASL11A, SIK1, CAMKK1, GNL3, PFKP	0.3965
GOTERM_CC_FAT	GO:0005954 ~ calcium- and calmodulin dependent protein kinase complex	2	0.0259	CAMK1G, CAMK2G	0.9773
GOTERM_BP_FAT	GO:0000185~activation of MAPKKK activity	2	0.0289	GADD45B, GADD45G	0.8292
UP_SEQ_FEATURE	Region of interest: Autoinhibitory domain	2	0.0307	CAMK1G, CAMKK1	0.7256
UP_SEQ_FEATURE	Repeat:WD 5	5	0.0330	CORO6, STRN3, WDR91, TLE3, PAK1IP1	0.7181
GOTERM_MF_FAT	GO:0004674~protein serine/threonine kinase activity	7	0.0337	LIMK1, CAMK1G, MARK1, CAMK2G, SIK1, CAMKK1, MAP3K6	0.8912
GOTERM_BP_FAT	GO:0009649~entrainment of circadian clock	2	0.0345	BHLHE40, PER1	0.8588
GOTERM_BP_FAT	GO:0048514~blood vessel morphogenesis	5	0.0349	JUN, NOTCH4, SCG2, ADRA1B, PLAT	0.8407
INTERPRO	IPR002087:Anti-prolifera tive protein	2	0.0392	TOB2, BTG2	0.7450

GOTERM_BP_FAT	GO:0042127~regulation of cell proliferation	10	0.0394	TESC, JUN, TOB2, NOTCH4, SCG2, BTG2, BAI1, SESN1, GNL3, MARCKSL1	0.8563
SP_PIR_KEYWORDS	ATP-binding	13	0.0395	CAMK1G, RTEL1, MARK1, CAMK2G, GNE, TYRO3, MAP3K6, LIMK1, CNKSR3, KIFC2, SIK1, CAMKK1, PFKP	0.5097
INTERPRO	IPR019782: WD40 repeat 2	5	0.0404	CORO6, STRN3, WDR91, TLE3, PAK1IP1	0.7134
UP_SEQ_FEATURE	Repeat: WD 4	5	0.0415	CORO6, STRN3, WDR91, TLE3, PAK1IP1	0.7695
KEGG_PATHWAY	hsa04010:MAPK signaling pathway	6	0.0423	DUSP1, JUN, GADD45B, GADD45G, MAPK8IP1, MAP3K6	0.5539
SMART	SM00099: btg1	2	0.0431	TOB2, BTG2	0.5116
UP_TISSUE	Fetal lung	3	0.0453	RTEL1, PLAT, SIK1	0.8473
SP_PIR_KEYWORDS	Apoptosis	6	0.0470	MRPL41, GADD45B, GADD45G, RTEL1, RASSF5, FEM1B	0.5390
INTERPRO	IPR017986: WD40 repeat, region	5	0.0473	CORO6, STRN3, WDR91, TLE3, PAK1IP1	0.7332
GENETIC_ASSOCIATION_DB_DISEASE	Alzheimer's Disease	5	0.0480	CYP46A1, NOTCH4, CAMK2G, PLAT, MAPK8IP1	0.9503
UP_SEQ_FEATURE	Repeat: WD 3	5	0.0490	CORO6, STRN3, WDR91, TLE3, PAK1IP1	0.7988
INTERPRO	IPR019781: WD40 repeat, subgroup	5	0.0497	CORO6, STRN3, WDR91, TLE3, PAK1IP1	0.7177
SP_PIR_KEYWORDS	Alternative splicing	48	0.0510	PDE4B, PITPNM1, NOTCH4, CAMK1G, RTEL1, GORAB, PER1, NUMB, IQSEC3, LIPE, RASSF5, CREM, GPR162, ZMYND8, LIMK1, CNKSR3, ARTN, SMOX, KCNH1, PLAT, DSCAM, WDR91, INPP4A, UPP1, TTC22, PLEKHG3, BEND5, GRIK5, TESC, CORO6, PMEPA1, STRN3, MARK1, CAMK2G, SESN1, RIMBP2, CHPF2, MAP3K6, DMP1, PLA1A, SEC61A2, PHRF1, SLC35F4, CAMKK1, CMIP, GNL3, TLE3, SORBS2	0.5383

SP_PIR_KEYWORDS	Transferase	13	0.0541	LIMK1, HS3ST2, CAMK1G, MARK1, CAMK2G, SIK1, GNE, CAMKK1, UPP1, TYRO3, PFKP, CHPF2, MAP3K6	0.5311
UP_SEQ_FEATURE	Repeat: WD 1	5	0.0541	CORO6, STRN3, WDR91, TLE3, PAK1IP1	0.8079
UP_SEQ_FEATURE	Repeat: WD 2	5	0.0541	CORO6, STRN3, WDR91, TLE3, PAK1IP1	0.8079
SP_PIR_KEYWORDS	WD repeat	5	0.0543	CORO6, STRN3, WDR91, TLE3, PAK1IP1	0.5062
GOTERM_BP_FAT	GO:0000904~cell morphogenesis involved in differentiation	5	0.0544	NOTCH4, NUMB, DSCAM, BAI1, RTN4RL2	0.9203
GOTERM_BP_FAT	GO:0001568~blood vessel development	5	0.0550	JUN, NOTCH4, SCG2, ADRA1B, PLAT	0.9103
UP_SEQ_FEATURE	Compositionally biased region: Gly/Pro-rich	2	0.0555	TLE3, CHPF2	0.7942
GOTERM_BP_FAT	GO:0001944~vasculature development	5	0.0591	JUN, NOTCH4, SCG2, ADRA1B, PLAT	0.9138
INTERPRO	IPR004038: Ribosomal protein L7Ae/L30e/S12e/Gadd45	2	0.0610	GADD45B, GADD45G	0.7611
GOTERM_BP_FAT	GO:0006595~polyamine metabolic process	2	0.0624	AZIN1, SMOX	0.9142
GOTERM_BP_FAT	GO:0045637~regulation of myeloid cell differentiation	3	0.0630	TESC, JUN, TOB2	0.9053
GOTERM_BP_FAT	GO:0009628~response to abiotic stimulus	6	0.0639	DUSP1, JUN, PITPNM1, ADRA1B, BTG2, CIRBP	0.8973
INTERPRO	IPR001680:WD40 repeat	5	0.0657	CORO6, STRN3, WDR91, TLE3, PAK1IP1	0.7601
GOTERM_BP_FAT	GO:0030522~intracellular	3	0.0711	KLF9, PMEPA1, CREM	0.9117

	receptor-mediated signaling pathway				
BIOCARTA	h_dreampathway: Repression of Pain Sensation by the Transcriptional Regulator DREAM	2	0.0729	JUN, CREM	0.9901
INTERPRO	IPR019775: WD40 repeat, conserved site	5	0.0739	CORO6, STRN3, WDR91, TLE3, PAK1IP1	0.7760
UP_SEQ_FEATURE	Splice variant	47	0.0788	PDE4B, PITPNM1, NOTCH4, CAMK1G, RTEL1, GORAB, NUMB, IQSEC3, LIPE, RASSF5, CREM, GPR162, ZMYND8, LIMK1, CNKSR3, ARTN, SMOX, KCNH1, PLAT, DSCAM, WDR91, INPP4A, UPP1, TTC22, PLEKHG3, BEND5, GRIK5, TESC, CORO6, PMEPA1, STRN3, MARK1, CAMK2G, SESN1, RIMBP2, CHPF2, MAP3K6, DMP1, PLA1A, SEC61A2, PHRF1, SLC35F4, CAMKK1, CMIP, GNL3, TLE3, SORBS2	0.8812
GOTERM_BP_FAT	GO:0051094~positive regulation of developmental process	5	0.0795	TESC, JUN, LIMK1, TOB2, NUMB	0.9263
GOTERM_CC_FAT	GO:0005625~soluble fraction	5	0.0816	PDE4B, PCSK2, RTEL1, CRHBP, PFKP	0.9978
SP_PIR_KEYWORDS	Ubl conjugation	7	0.0829	BHLHE40, NUMB, PER1, RIMBP2, MAPK8IP1, ADRM1, SORBS2	0.6399
GOTERM_MF_FAT	GO:0046983~protein dimerization activity	7	0.0839	JUN, LIMK1, NOTCH4, MAFK, NFIL3, NPAS4, CREM	0.9893
SMART	SM00320: WD40	5	0.0852	CORO6, STRN3, WDR91, TLE3, PAK1IP1	0.6859
GOTERM_BP_FAT	GO:0006355~regulation	16	0.0873	TESC, JUN, KLF9, STRN3, NOTCH4, BHLHE40, MAFK, PER1,	0.9365

	of transcription, DNA-dependent			TCF25, NPAS4, CREM, KCNH1, SIK1, NFIL3, MAPK8IP1, TLE3	
GOTERM_BP_FAT	GO:0007405~neuroblast proliferation	2	0.0895	ARTN, NUMB	0.9338
GOTERM_BP_FAT	GO:0006469~negative regulation of protein kinase activity	3	0.0917	GADD45B, GADD45G, MAPK8IP1	0.9314
INTERPRO	IPR015943: WD40/YVTN repeat-like	5	0.0925	CORO6, STRN3, WDR91, TLE3, PAK1IP1	0.8290
GENETIC_ASSOCIATION_DB_DISEASE_CLASS	PSYCH	7	0.0936	PDE4B, GRIK5, NOTCH4, PLAT, PER1, CRHBP, CREM	0.6608
UP_SEQ_FEATURE	Domain: PAS 1	2	0.0940	PER1, NPAS4	0.9102
UP_SEQ_FEATURE	Domain: SH3 3	2	0.0940	RIMBP2, SORBS2	0.9102
UP_SEQ_FEATURE	Domain: PAS 2	2	0.0940	PER1, NPAS4	0.9102
GOTERM_BP_FAT	GO:0033673~negative regulation of kinase activity	3	0.0971	GADD45B, GADD45G, MAPK8IP1	0.9354
INTERPRO	IPR013655: PAS fold-3	2	0.0979	PER1, NPAS4	0.8275

Table S4 Function cluster of top 100 mRNAs

Category	Term	Count	P-Value	Genes	Benjamini
**Annotation Cluster 1**<br>**Enrichment Score: 1.8874016802736022**					
**Category**	**Term**	**Count**	**P-Value**	**Genes**	**Benjamini**
UP_SEQ_FEATURE	Domain: PAC	3	0.0080	KCNH1, PER1, NPAS4	0.5636
INTERPRO	IPR000014: PAS	3	0.0151	KCNH1, PER1, NPAS4	0.8755
SMART	SM00091: PAS	3	0.0181	KCNH1, PER1, NPAS4	0.4468
**Annotation Cluster 2**<br>**Enrichment Score: 1.6684704277730393**					
**Category**	**Term**	**Count**	**P-Value**	**Genes**	**Benjamini**
UP_SEQ_FEATURE	Region of interest: Calmodulin-binding	5	0.0002	STRN3, CAMK1G, CAMK2G, KCNH1, CAMKK1	0.0753
SP_PIR_KEYWORDS	Calmodulin-binding	6	0.0004	STRN3, CAMK1G, CAMK2G, KCNH1, CAMKK1, MARCKSL1	0.0339
SP_PIR_KEYWORDS	Kinase	12	0.0008	ZMYND8, LIMK1, CAMK1G, MARK1, CAMK2G, SIK1, GNE, CAMKK1, MAPK8IP1, TYRO3, PFKP, MAP3K6	0.0472
GOTERM_MF_FAT	GO:0005516~calmodulin binding	6	0.0011	STRN3, CAMK1G, CAMK2G, KCNH1, CAMKK1, MARCKSL1	0.2543
GOTERM_BP_FAT	GO:0006468~protein amino acid phosphorylation	12	0.0016	GADD45B, LIMK1, CAMK1G, GADD45G, MARK1, CAMK2G, SIK1, CAMKK1, MAPK8IP1, LIPE, TYRO3, MAP3K6	0.2111
UP_SEQ_FEATURE	Nucleotide phosphate-binding region: ATP	13	0.0040	CAMK1G, RTEL1, MARK1, CAMK2G, GNE, TYRO3, MAP3K6, LIMK1, CNKSR3, KIFC2, SIK1, CAMKK1, PFKP	0.4293
GOTERM_MF_FAT	GO:0004683~calmodulin-depend	3	0.0055	CAMK1G, CAMK2G, CAMKK1	0.5136

	ent protein kinase activity				
GOTERM_BP_FAT	GO:0016310~phosphorylation	12	0.0066	GADD45B, LIMK1, CAMK1G, GADD45G, MARK1, CAMK2G, SIK1, CAMKK1, MAPK8IP1, LIPE, TYRO3, MAP3K6	0.4937
GOTERM_BP_FAT	GO:0006793~phosphorus metabolic process	13	0.0105	DUSP1, GADD45B, LIMK1, CAMK1G, GADD45G, MARK1, CAMK2G, SIK1, CAMKK1, MAPK8IP1, LIPE, TYRO3, MAP3K6	0.6137
GOTERM_BP_FAT	GO:0006796~phosphate metabolic process	13	0.0105	DUSP1, GADD45B, LIMK1, CAMK1G, GADD45G, MARK1, CAMK2G, SIK1, CAMKK1, MAPK8IP1, LIPE, TYRO3, MAP3K6	0.6137
UP_SEQ_FEATURE	Domain: Protein kinase	8	0.0110	LIMK1, CAMK1G, MARK1, CAMK2G, SIK1, CAMKK1, TYRO3, MAP3K6	0.6009
SP_PIR_KEYWORDS	Serine/threonine-protein kinase	7	0.0139	LIMK1, CAMK1G, MARK1, CAMK2G, SIK1, CAMKK1, MAP3K6	0.3387
INTERPRO	IPR017441: Protein kinase, ATP binding site	8	0.0152	LIMK1, CAMK1G, MARK1, CAMK2G, SIK1, CAMKK1, TYRO3, MAP3K6	0.7530
INTERPRO	IPR008271: Serine/threonine protein kinase, active site	7	0.0158	LIMK1, CAMK1G, MARK1, CAMK2G, SIK1, CAMKK1, MAP3K6	0.6622
INTERPRO	IPR002290: Serine/threonine protein kinase	6	0.0163	CAMK1G, MARK1, CAMK2G, SIK1, CAMKK1, MAP3K6	0.5926
INTERPRO	IPR017442: Serine/threonine protein kinase-related	7	0.0168	LIMK1, CAMK1G, MARK1, CAMK2G, SIK1, CAMKK1, MAP3K6	0.5372
INTERPRO	IPR000719: Protein kinase, core	8	0.0190	LIMK1, CAMK1G, MARK1, CAMK2G, SIK1, CAMKK1, TYRO3, MAP3K6	0.5276
GOTERM_MF_FAT	GO:0004672~protein kinase activity	9	0.0201	LIMK1, CAMK1G, MARK1, CAMK2G, KCNH1, SIK1, CAMKK1, TYRO3, MAP3K6	0.8263

UP_SEQ_FEATURE	Active site: Proton acceptor	9	0.0209	CAMK1G, ALDH1A1, MARK1, CAMK2G, SIK1, CAMKK1, TYRO3, PFKP, MAP3K6	0.6655
UP_SEQ_FEATURE	Binding site: ATP	8	0.0225	LIMK1, CAMK1G, MARK1, CAMK2G, SIK1, CAMKK1, TYRO3, MAP3K6	0.6503
SMART	SM00220:S_TKc	6	0.0228	CAMK1G, MARK1, CAMK2G, SIK1, CAMKK1, MAP3K6	0.3932
SP_PIR_KEYWORDS	Nucleotide-binding	16	0.0254	CAMK1G, RTEL1, CRY1, MARK1, CAMK2G, GNE, TYRO3, MAP3K6, LIMK1, CNKSR3, KIFC2, RASL11A, SIK1, CAMKK1, GNL3, PFKP	0.3965
GOTERM_MF_FAT	GO:0004674~protein serine/threonine kinase activity	7	0.0337	LIMK1, CAMK1G, MARK1, CAMK2G, SIK1, CAMKK1, MAP3K6	0.8912
SP_PIR_KEYWORDS	ATP-binding	13	0.0395	CAMK1G, RTEL1, MARK1, CAMK2G, GNE, TYRO3, MAP3K6, LIMK1, CNKSR3, KIFC2, SIK1, CAMKK1, PFKP	0.5097
SP_PIR_KEYWORDS	Transferase	13	0.0541	LIMK1, HS3ST2, CAMK1G, MARK1, CAMK2G, SIK1, GNE, CAMKK1, UPP1, TYRO3, PFKP, CHPF2, MAP3K6	0.5311
GOTERM_MF_FAT	GO:0005524~ATP binding	13	0.1213	CAMK1G, RTEL1, MARK1, CAMK2G, GNE, TYRO3, MAP3K6, LIMK1, CNKSR3, KIFC2, SIK1, CAMKK1, PFKP	0.9962
GOTERM_MF_FAT	GO:0032559~adenyl ribonucleotide binding	13	0.1303	CAMK1G, RTEL1, MARK1, CAMK2G, GNE, TYRO3, MAP3K6, LIMK1, CNKSR3, KIFC2, SIK1, CAMKK1, PFKP	0.9943
GOTERM_MF_FAT	GO:0032555~purine ribonucleotide binding	15	0.1429	CAMK1G, RTEL1, MARK1, CAMK2G, GNE, TYRO3, MAP3K6, LIMK1, CNKSR3, KIFC2, SIK1, RASL11A, CAMKK1, GNL3, PFKP	0.9932
GOTERM_MF_FAT	GO:0032553~ribonucleotide	15	0.1429	CAMK1G, RTEL1, MARK1, CAMK2G, GNE, TYRO3,	0.9932

	binding			MAP3K6, LIMK1, CNKSR3, KIFC2, SIK1, RASL11A, CAMKK1, GNL3, PFKP	
GOTERM_MF_FAT	GO:0030554~adenyl nucleotide binding	13	0.1703	CAMK1G, RTEL1, MARK1, CAMK2G, GNE, TYRO3, MAP3K6, LIMK1, CNKSR3, KIFC2, SIK1, CAMKK1, PFKP	0.9954
GOTERM_MF_FAT	GO:0017076~purine nucleotide binding	15	0.1822	CAMK1G, RTEL1, MARK1, CAMK2G, GNE, TYRO3, MAP3K6, LIMK1, CNKSR3, KIFC2, SIK1, RASL11A, CAMKK1, GNL3, PFKP	0.9912
GOTERM_MF_FAT	GO:0001883~purine nucleoside binding	13	0.1834	CAMK1G, RTEL1, MARK1, CAMK2G, GNE, TYRO3, MAP3K6, LIMK1, CNKSR3, KIFC2, SIK1, CAMKK1, PFKP	0.9874
GOTERM_MF_FAT	GO:0000166~nucleotide binding	17	0.1839	CAMK1G, RTEL1, CRY1, MARK1, CAMK2G, GNE, TYRO3, MAP3K6, LIMK1, CNKSR3, KIFC2, CIRBP, RASL11A, SIK1, CAMKK1, GNL3, PFKP	0.9826
GOTERM_MF_FAT	GO:0001882~nucleoside binding	13	0.1896	CAMK1G, RTEL1, MARK1, CAMK2G, GNE, TYRO3, MAP3K6, LIMK1, CNKSR3, KIFC2, SIK1, CAMKK1, PFKP	0.9795
GOTERM_MF_FAT	GO:0000287~magnesium ion binding	5	0.2492	TESC, MARK1, SIK1, PFKP, MAP3K6	0.9903
SP_PIR_KEYWORDS	magnesium	4	0.3983	MARK1, SIK1, PFKP, MAP3K6	0.9550
**Annotation Cluster 3**<br>**Enrichment Score: 1.2138199962269758**					
**Category**	**Term**	**Count**	**P-Value**	**Genes**	**Benjamini**
UP_SEQ_FEATURE	Repeat: WD 5	5	0.0330	CORO6, STRN3, WDR91, TLE3, PAK1IP1	0.7516
INTERPRO	IPR019782: WD40 repeat 2	5	0.0404	CORO6, STRN3, WDR91, TLE3, PAK1IP1	0.7548
UP_SEQ_FEATURE	Repeat: WD 4	5	0.0415	CORO6, STRN3, WDR91, TLE3, PAK1IP1	0.7983

INTERPRO	IPR017986: WD40 repeat, region	5	0.0473	CORO6, STRN3, WDR91, TLE3, PAK1IP1	0.7697
UP_SEQ_FEATURE	Repeat: WD 3	5	0.0490	CORO6, STRN3, WDR91, TLE3, PAK1IP1	0.8239
INTERPRO	IPR019781: WD40 repeat, subgroup	5	0.0497	CORO6, STRN3, WDR91, TLE3, PAK1IP1	0.7512
UP_SEQ_FEATURE	Repeat: WD 2	5	0.0541	CORO6, STRN3, WDR91, TLE3, PAK1IP1	0.8308
UP_SEQ_FEATURE	Repeat: WD 1	5	0.0541	CORO6, STRN3, WDR91, TLE3, PAK1IP1	0.8308
SP_PIR_KEYWORDS	WD repeat	5	0.0543	CORO6, STRN3, WDR91, TLE3, PAK1IP1	0.5062
INTERPRO	IPR001680: WD40 repeat	5	0.0657	CORO6, STRN3, WDR91, TLE3, PAK1IP1	0.8149
INTERPRO	IPR019775: WD40 repeat, conserved site	5	0.0739	CORO6, STRN3, WDR91, TLE3, PAK1IP1	0.8255
SMART	SM00320: WD40	5	0.0852	CORO6, STRN3, WDR91, TLE3, PAK1IP1	0.7648
INTERPRO	IPR015943: WD40/YVTN repeat-like	5	0.0925	CORO6, STRN3, WDR91, TLE3, PAK1IP1	0.8697
UP_SEQ_FEATURE	Repeat: WD 6	3	0.2622	STRN3, WDR91, TLE3	0.9922
**Annotation Cluster 4**<br>**Enrichment Score: 1.1180821627432336**					
**Category**	**Term**	**Count**	**P-Value**	**Genes**	**Benjamini**
GOTERM_BP_FAT	GO:0007243~protein kinase cascade	9	0.0014	GADD45B, NOTCH4, SCG2, ADRA1B, GADD45G, MARK1, SIK1, MAPK8IP1, MAP3K6	0.2232
SP_PIR_KEYWORDS	Differentiation	5	0.2126	GADD45B, NOTCH4, GADD45G, SIK1, CREM	0.9289
GOTERM_BP_FAT	GO:0051726~regulation of cell cycle	4	0.3049	JUN, GADD45B, GADD45G, SIK1	0.9893
SP_PIR_KEYWORDS	Developmental protein	6	0.3730	GADD45B, NOTCH4, GADD45G, SIK1, NUMB, CREM	0.9531
**Annotation Cluster 5**<br>**Enrichment Score: 0.9373017376402812**					
**Category**	**Term**	**Count**	**P-Value**	**Genes**	**Benjamini**
UP_SEQ_FEATURE	DNA-binding region:Basic motif	6	0.0015	JUN, BHLHE40, MAFK, NFIL3, NPAS4, CREM	0.2688
INTERPRO	IPR004827: Basic-leucine zipper	4	0.0034	JUN, MAFK, NFIL3, CREM	0.6080

	(bZIP) transcription factor				
SMART	SM00338: BRLZ	4	0.0044	JUN, MAFK, NFIL3, CREM	0.2493
UP_SEQ_FEATURE	Domain: Leucine-zipper	4	0.0195	JUN, MAFK, NFIL3, CREM	0.6892
SP_PIR_KEYWORDS	Repressor	7	0.0249	CRY1, MAFK, CIRBP, TCF25, NFIL3, TLE3, CREM	0.4276
GOTERM_MF_FAT	GO:0046983~protein dimerization activity	7	0.0839	JUN, LIMK1, NOTCH4, MAFK, NFIL3, NPAS4, CREM	0.9893
GOTERM_BP_FAT	GO:0006355~regulation of transcription, DNA-dependent	16	0.0873	TESC, JUN, KLF9, STRN3, NOTCH4, BHLHE40, MAFK, PER1, TCF25, NPAS4, CREM, KCNH1, SIK1, NFIL3, MAPK8IP1, TLE3	0.9572
GOTERM_BP_FAT	GO:0051252~regulation of RNA metabolic process	16	0.1011	TESC, JUN, KLF9, STRN3, NOTCH4, BHLHE40, MAFK, PER1, TCF25, NPAS4, CREM, KCNH1, SIK1, NFIL3, MAPK8IP1, TLE3	0.9599
GOTERM_MF_FAT	GO:0003700~transcription factor activity	9	0.1803	KLF9, JUN, STRN3, CCRN4L, BHLHE40, MAFK, TCF25, NFIL3, CREM	0.9942
GOTERM_BP_FAT	GO:0006350~transcription	16	0.2378	JUN, KLF9, NOTCH4, BTG2, BHLHE40, CRY1, MAFK, PER1, TCF25, NPAS4, CREM, PHRF1, CCRN4L, NFIL3, TLE3, ADRM1	0.9861
GOTERM_MF_FAT	GO:0030528~transcription regulator activity	11	0.3427	KLF9, JUN, STRN3, CCRN4L, BHLHE40, SIK1, MAFK, TCF25, NFIL3, NPAS4, CREM	0.9956
GOTERM_BP_FAT	GO:0045449~regulation of transcription	18	0.3446	TESC, JUN, KLF9, STRN3, NOTCH4, BTG2, BHLHE40, CRY1, MAFK, PER1, TCF25, NPAS4, CREM, KCNH1, SIK1, NFIL3, MAPK8IP1, TLE3	0.9857
SP_PIR_KEYWORDS	Transcription regulation	13	0.3485	JUN, KLF9, NOTCH4, BTG2, CRY1, BHLHE40, MAFK, PER1, TCF25, NPAS4, CREM, NFIL3, TLE3	0.9682

SP_PIR_KEYWORDS	Nucleus	25	0.3550	JUN, KLF9, NOTCH4, RTEL1, BHLHE40, PER1, ANXA11, CREM, CIRBP, NFIL3, MAPK8IP1, PAK1IP1, CRY1, MAFK, TCF25, SESN1, NPAS4, DMP1, ASPA, SIK1, CAMKK1, GNL3, CMIP, TLE3, ADRM1	0.9658
SP_PIR_KEYWORDS	Transcription	13	0.3775	JUN, KLF9, NOTCH4, BTG2, CRY1, BHLHE40, MAFK, PER1, TCF25, NPAS4, CREM, NFIL3, TLE3	0.9500
GOTERM_BP_FAT	GO:0006357~regulation of transcription from RNA polymerase II promoter	6	0.4204	KLF9, JUN, STRN3, SIK1, TCF25, NPAS4	0.9863
GOTERM_MF_FAT	GO:0008134~transcription factor binding	4	0.5551	JUN, TOB2, NFIL3, CREM	0.9996
GOTERM_MF_FAT	GO:0043565~sequence-specific DNA binding	4	0.6700	JUN, MAFK, NFIL3, CREM	0.9998
SP_PIR_KEYWORDS	DNA-binding	9	0.7571	KLF9, JUN, RTEL1, BHLHE40, MAFK, TCF25, NFIL3, NPAS4, CREM	0.9957
GOTERM_MF_FAT	GO:0003677~DNA binding	12	0.7838	KLF9, JUN, STRN3, CCRN4L, RTEL1, BHLHE40, CRY1, MAFK, TCF25, NFIL3, NPAS4, CREM	0.9999
**Annotation Cluster 6**<br>**Enrichment Score: 0.8511405646592396**					
**Category**	**Term**	**Count**	**P-Value**	**Genes**	**Benjamini**
GOTERM_BP_FAT	GO:0033554~cellular response to stress	9	0.0172	JUN, BTG2, GADD45G, RTEL1, CRY1, SESN1, MAPK8IP1, MAP3K6, RTN4RL2	0.7527
GOTERM_BP_FAT	GO:0006974~response to DNA damage stimulus	5	0.1736	BTG2, GADD45G, RTEL1, CRY1, SESN1	0.9710

GOTERM_BP_FAT	GO:0006281~DNA repair	4	0.2306	BTG2, GADD45G, RTEL1, CRY1	0.9853
GOTERM_BP_FAT	GO:0006259~DNA metabolic process	4	0.5714	BTG2, GADD45G, RTEL1, CRY1	0.9964
**Annotation Cluster 7**<br>**Enrichment Score: 0.822982321837618**					
**Category**	**Term**	**Count**	**P-Value**	**Genes**	**Benjamini**
UP_SEQ_FEATURE	DNA-binding region: Basic motif	6	0.0015	JUN, BHLHE40, MAFK, NFIL3, NPAS4, CREM	0.2688
GOTERM_MF_FAT	GO:0046983~protein dimerization activity	7	0.0839	JUN, LIMK1, NOTCH4, MAFK, NFIL3, NPAS4, CREM	0.9893
SP_PIR_KEYWORDS	Activator	5	0.2795	NOTCH4, CIRBP, NFIL3, NPAS4, CREM	0.9671
GOTERM_CC_FAT	GO:0005667~transcription factor complex	3	0.3016	JUN, NPAS4, CREM	0.9943
GOTERM_CC_FAT	GO:0005654~nucleoplasm	7	0.3156	JUN, STRN3, CIRBP, GNL3, NPAS4, ANXA11, CREM	0.9798
GOTERM_CC_FAT	GO:0031981~nuclear lumen	8	0.6490	JUN, STRN3, CIRBP, GNL3, NPAS4, ANXA11, PAK1IP1, CREM	0.9945
GOTERM_CC_FAT	GO:0044451~nucleoplasm part	3	0.7943	JUN, NPAS4, CREM	0.9979
**Annotation Cluster 8**<br>**Enrichment Score: 0.7986265184403722**					
**Category**	**Term**	**Count**	**P-Value**	**Genes**	**Benjamini**
GOTERM_BP_FAT	GO:0009628~response to abiotic stimulus	6	0.0639	DUSP1, JUN, PITPNM1, ADRA1B, BTG2, CIRBP	0.9297
GOTERM_BP_FAT	GO:0009416~response to light stimulus	3	0.1927	DUSP1, PITPNM1, ADRA1B	0.9772
GOTERM_BP_FAT	GO:0009314~response to radiation	3	0.3264	DUSP1, PITPNM1, ADRA1B	0.9893
**Annotation Cluster 9**<br>**Enrichment Score: 0.7685351669695512**					
**Category**	**Term**	**Count**	**P-Value**	**Genes**	**Benjamini**

SP_PIR_KEYWORDS	Apoptosis	6	0.0470	MRPL41, GADD45B, GADD45G, RTEL1, RASSF5, FEM1B	0.5390
GOTERM_BP_FAT	GO:0006915~apoptosis	7	0.1393	MRPL41, JUN, GADD45B, GADD45G, RTEL1, RASSF5, FEM1B	0.9664
GOTERM_BP_FAT	GO:0012501~programmed cell death	7	0.1464	MRPL41, JUN, GADD45B, GADD45G, RTEL1, RASSF5, FEM1B	0.9689
UP_TISSUE	Colon carcinoma	3	0.2380	GADD45B, GADD45G, RTEL1	0.9745
GOTERM_BP_FAT	GO:0008219~cell death	7	0.2426	MRPL41, JUN, GADD45B, GADD45G, RTEL1, RASSF5, FEM1B	0.9862
GOTERM_BP_FAT	GO:0016265~death	7	0.2475	MRPL41, JUN, GADD45B, GADD45G, RTEL1, RASSF5, FEM1B	0.9850
GOTERM_BP_FAT	GO:0051726~regulation of cell cycle	4	0.3049	JUN, GADD45B, GADD45G, SIK1	0.9893

Annotation Cluster 10
Enrichment Score: 0.7485071613471423

**Category**	**Term**	**Count**	**P-Value**	**Genes**	**Benjamini**
GOTERM_BP_FAT	GO:0048514~blood vessel morphogenesis	5	0.0349	JUN, NOTCH4, SCG2, ADRA1B, PLAT	0.8827
GOTERM_BP_FAT	GO:0001568~blood vessel development	5	0.0550	JUN, NOTCH4, SCG2, ADRA1B, PLAT	0.9349
GOTERM_BP_FAT	GO:0001944~vasculature development	5	0.0591	JUN, NOTCH4, SCG2, ADRA1B, PLAT	0.9366
GOTERM_BP_FAT	GO:0007611~learning or memory	3	0.1372	JUN, ADRA1B, CRHBP	0.9716
GOTERM_BP_FAT	GO:0007610~behavior	6	0.1389	JUN, SCG2, ADRA1B, PGLYRP1, DSCAM, CRHBP	0.9696
GOTERM_BP_FAT	GO:0001525~angiogenesis	3	0.2140	JUN, NOTCH4, SCG2	0.9827
GOTERM_BP_FAT	GO:0008284~positive regulation of cell proliferation	4	0.4369	JUN, NOTCH4, SCG2, MARCKSL1	0.9868
GOTERM_BP_FAT	GO:0050877~neurological system process	5	0.9314	JUN, GRIK5, ADRA1B, PLAT, CRHBP	1
GOTERM_BP_FAT	GO:0050890~cognition	3	0.9728	JUN, ADRA1B, CRHBP	1

**Annotation Cluster 11**<br>**Enrichment Score: 0.7371450203624088**					
**Category**	**Term**	**Count**	**P-Value**	**Genes**	**Benjamini**
GOTERM_BP_FAT	GO:0007243~protein kinase cascade	9	0.0014	GADD45B, NOTCH4, SCG2, ADRA1B, GADD45G, MARK1, SIK1, MAPK8IP1, MAP3K6	0.2232
GOTERM_BP_FAT	GO:0033554~cellular response to stress	9	0.0172	JUN, BTG2, GADD45G, RTEL1, CRY1, SESN1, MAPK8IP1, MAP3K6, RTN4RL2	0.7527
GOTERM_BP_FAT	GO:0000165~MAPKKK cascade	5	0.0226	GADD45B, SCG2, GADD45G, MAPK8IP1, MAP3K6	0.7777
KEGG_PATHWAY	hsa04010:MAPK signaling pathway	6	0.0423	DUSP1, JUN, GADD45B, GADD45G, MAPK8IP1, MAP3K6	0.5539
GOTERM_BP_FAT	GO:0006469~negative regulation of protein kinase activity	3	0.0917	GADD45B, GADD45G, MAPK8IP1	0.9578
GOTERM_BP_FAT	GO:0033673~negative regulation of kinase activity	3	0.0971	GADD45B, GADD45G, MAPK8IP1	0.9599
GOTERM_BP_FAT	GO:0051348~negative regulation of transferase activity	3	0.1082	GADD45B, GADD45G, MAPK8IP1	0.9637
GOTERM_BP_FAT	GO:0031399~regulation of protein modification process	4	0.2477	JUN, MAPK8IP1, MAP3K6, FEM1B	0.9838
GOTERM_BP_FAT	GO:0001932~regulation of protein amino acid phosphorylation	3	0.2680	JUN, MAPK8IP1, MAP3K6	0.9848
GOTERM_BP_FAT	GO:0042325~regulation of phosphorylation	5	0.2893	JUN, GADD45B, GADD45G, MAPK8IP1, MAP3K6	0.9879
GOTERM_BP_FAT	GO:0051174~regulation of phosphorus metabolic process	5	0.3144	JUN, GADD45B, GADD45G, MAPK8IP1, MAP3K6	0.9887
GOTERM_BP_FAT	GO:0019220~regulation of phosphate metabolic process	5	0.3144	JUN, GADD45B, GADD45G, MAPK8IP1, MAP3K6	0.9887

GOTERM_BP_FAT	GO:0045859~regulation of protein kinase activity	4	0.3273	GADD45B, GADD45G, MAPK8IP1, MAP3K6	0.9887
GOTERM_BP_FAT	GO:0043549~regulation of kinase activity	4	0.3466	GADD45B, GADD45G, MAPK8IP1, MAP3K6	0.9844
GOTERM_BP_FAT	GO:0051338~regulation of transferase activity	4	0.3706	GADD45B, GADD45G, MAPK8IP1, MAP3K6	0.9841
GOTERM_BP_FAT	GO:0045860~positive regulation of protein kinase activity	3	0.3752	GADD45B, GADD45G, MAP3K6	0.9826
GOTERM_BP_FAT	GO:0033674~positive regulation of kinase activity	3	0.3918	GADD45B, GADD45G, MAP3K6	0.9841
GOTERM_BP_FAT	GO:0051347~positive regulation of transferase activity	3	0.4103	GADD45B, GADD45G, MAP3K6	0.9864
GOTERM_BP_FAT	GO:0043086~negative regulation of catalytic activity	3	0.4834	GADD45B, GADD45G, MAPK8IP1	0.9916
GOTERM_BP_FAT	GO:0032268~regulation of cellular protein metabolic process	4	0.5267	JUN, MAPK8IP1, MAP3K6, FEM1B	0.9945
GOTERM_BP_FAT	GO:0044092~negative regulation of molecular function	3	0.5845	GADD45B, GADD45G, MAPK8IP1	0.9966
GOTERM_BP_FAT	GO:0043085~positive regulation of catalytic activity	3	0.8129	GADD45B, GADD45G, MAP3K6	0.9999
GOTERM_BP_FAT	GO:0044093~positive regulation of molecular function	3	0.8623	GADD45B, GADD45G, MAP3K6	1
**Annotation Cluster 12**<br>**Enrichment Score: 0.6935395708847507**					
**Category**	**Term**	**Count**	**P-Value**	**Genes**	**Benjamini**
GOTERM_BP_FAT	GO:0000904~cell morphogenesis involved in	5	0.0544	NOTCH4, NUMB, DSCAM, BAI1, RTN4RL2	0.9445

	differentiation				
GOTERM_BP_FAT	GO:0048667~cell morphogenesis involved in neuron differentiation	4	0.1231	NUMB, DSCAM, BAI1, RTN4RL2	0.9705
GOTERM_BP_FAT	GO:0048812~neuron projection morphogenesis	4	0.1283	NUMB, DSCAM, BAI1, RTN4RL2	0.9713
GOTERM_BP_FAT	GO:0000902~cell morphogenesis	5	0.1545	NOTCH4, NUMB, DSCAM, BAI1, RTN4RL2	0.9690
GOTERM_BP_FAT	GO:0048858~cell projection morphogenesis	4	0.1723	NUMB, DSCAM, BAI1, RTN4RL2	0.9727
GOTERM_BP_FAT	GO:0032990~cell part morphogenesis	4	0.1883	NUMB, DSCAM, BAI1, RTN4RL2	0.9771
GOTERM_BP_FAT	GO:0031175~neuron projection development	4	0.1883	NUMB, DSCAM, BAI1, RTN4RL2	0.9771
GOTERM_BP_FAT	GO:0032989~cellular component morphogenesis	5	0.2019	NOTCH4, NUMB, DSCAM, BAI1, RTN4RL2	0.9795
GOTERM_BP_FAT	GO:0030182~neuron differentiation	5	0.2530	BTG2, NUMB, DSCAM, BAI1, RTN4RL2	0.9828
GOTERM_CC_FAT	GO:0031226~intrinsic to plasma membrane	9	0.3031	NOTCH4, ADRA1B, KCNH1, NUMB, DSCAM, BAI1, TYRO3, ADRM1, RTN4RL2	0.9911
GOTERM_BP_FAT	GO:0007409~axonogenesis	3	0.3113	NUMB, BAI1, RTN4RL2	0.9889
GOTERM_BP_FAT	GO:0048666~neuron development	4	0.3177	NUMB, DSCAM, BAI1, RTN4RL2	0.9885
GOTERM_BP_FAT	GO:0030030~cell projection organization	4	0.3642	NUMB, DSCAM, BAI1, RTN4RL2	0.9834
GOTERM_CC_FAT	GO:0005887~integral to plasma membrane	8	0.4319	NOTCH4, ADRA1B, KCNH1, NUMB, DSCAM, BAI1, TYRO3, ADRM1	0.9938
**Annotation Cluster 13**<br>**Enrichment Score: 0.674203252406384**					
**Category**	**Term**	**Count**	**P-Value**	**Genes**	**Benjamini**
GOTERM_BP_FAT	GO:0006366~transcription from RNA polymerase II promoter	4	0.1567	CCRN4L, PHRF1, NFIL3, ADRM1	0.9675
GOTERM_BP_FAT	GO:0006351~transcription, DNA-dependent	4	0.2430	CCRN4L, PHRF1, NFIL3, ADRM1	0.9850

GOTERM_BP_FAT	GO:0032774~RNA biosynthetic process	4	0.2493	CCRN4L, PHRF1, NFIL3, ADRM1	0.9829
**Annotation Cluster 14**<br>**Enrichment Score: 0.6438870427234156**					
**Category**	**Term**	**Count**	**P-Value**	**Genes**	**Benjamini**
UP_SEQ_FEATURE	Domain: Fibronectin type-III 2	3	0.1459	DSCAM, RIMBP2, TYRO3	0.9787
UP_SEQ_FEATURE	Domain: Fibronectin type-III 1	3	0.1477	DSCAM, RIMBP2, TYRO3	0.9695
INTERPRO	IPR008957: Fibronectin, type III-like fold	3	0.2825	DSCAM, RIMBP2, TYRO3	0.9985
INTERPRO	IPR003961: Fibronectin, type III	3	0.2952	DSCAM, RIMBP2, TYRO3	0.9983
SMART	SM00060:FN3	3	0.3357	DSCAM, RIMBP2, TYRO3	0.9951
**Annotation Cluster 15**<br>**Enrichment Score: 0.6220131216467188**					
**Category**	**Term**	**Count**	**P-Value**	**Genes**	**Benjamini**
SP_PIR_KEYWORDS	Ubl conjugation	7	0.0829	BHLHE40, NUMB, PER1, RIMBP2, MAPK8IP1, ADRM1, SORBS2	0.6399
SP_PIR_KEYWORDS	SH3 domain	3	0.2942	RIMBP2, MAPK8IP1, SORBS2	0.9675
INTERPRO	IPR001452:Src homology-3 domain	3	0.3434	RIMBP2, MAPK8IP1, SORBS2	0.9976
SMART	SM00326:SH3	3	0.3881	RIMBP2, MAPK8IP1, SORBS2	0.9815
**Annotation Cluster 16**<br>**Enrichment Score: 0.6023790117034558**					
**Category**	**Term**	**Count**	**P-Value**	**Genes**	**Benjamini**
GOTERM_CC_FAT	GO:0030054~cell junction	6	0.1348	GRIK5, LIMK1, CNKSR3, BAI1, RIMBP2, SYT12	0.9990
GOTERM_CC_FAT	GO:0045202~synapse	4	0.2850	GRIK5, PLAT, RIMBP2, SYT12	0.9990
SP_PIR_KEYWORDS	Synapse	3	0.2999	GRIK5, RIMBP2, SYT12	0.9639
SP_PIR_KEYWORDS	Cell junction	4	0.3380	GRIK5, CNKSR3, RIMBP2, SYT12	0.9691

**Annotation Cluster 17**<br>**Enrichment Score: 0.556066340480945**					
**Category**	**Term**	**Count**	**P-Value**	**Genes**	**Benjamini**
GOTERM_BP_FAT	GO:0009628~response to abiotic stimulus	6	0.0639	DUSP1, JUN, PITPNM1, ADRA1B, BTG2, CIRBP	0.9297
GOTERM_BP_FAT	GO:0014070~response to organic cyclic substance	3	0.1573	JUN, ADRA1B, BTG2	0.9649
GOTERM_BP_FAT	GO:0009719~response to endogenous stimulus	4	0.4229	DUSP1, STRN3, ADRA1B, BTG2	0.9861
GOTERM_BP_FAT	GO:0010033~response to organic substance	5	0.6133	DUSP1, JUN, STRN3, ADRA1B, BTG2	0.9977
GOTERM_BP_FAT	GO:0009725~response to hormone stimulus	3	0.6361	DUSP1, STRN3, BTG2	0.9984
**Annotation Cluster 18**<br>**Enrichment Score: 0.5039578184612449**					
**Category**	**Term**	**Count**	**P-Value**	**Genes**	**Benjamini**
UP_SEQ_FEATURE	Domain: PH	4	0.1250	IQSEC3, CMIP, ADRM1, PLEKHG3	0.9687
INTERPRO	IPR001849: Pleckstrin homology	3	0.4706	IQSEC3, CMIP, PLEKHG3	0.9998
SMART	SM00233: PH	3	0.5230	IQSEC3, CMIP, PLEKHG3	0.9952
**Annotation Cluster 19**<br>**Enrichment Score: 0.5024514773525679**					
**Category**	**Term**	**Count**	**P-Value**	**Genes**	**Benjamini**
GOTERM_CC_FAT	GO:0000267~cell fraction	10	0.1111	PDE4B, PITPNM1, CYP46A1, PCSK2, ADRA1B, RTEL1, DSCAM, CRHBP, PFKP, ADRM1	0.9998
GOTERM_CC_FAT	GO:0005626~insoluble fraction	6	0.4511	PDE4B, PITPNM1, CYP46A1, ADRA1B, DSCAM, ADRM1	0.9918
GOTERM_CC_FAT	GO:0005624~membrane fraction	5	0.6201	PITPNM1, CYP46A1, ADRA1B, DSCAM, ADRM1	0.9931

**Annotation Cluster 20<br>Enrichment Score: 0.49517158876866874**					
**Category**	**Term**	**Count**	**P-Value**	**Genes**	**Benjamini**
GOTERM_MF_FAT	GO:0005083~small GTPase regulator activity	4	0.1998	JUN, ALDH1A1, IQSEC3, PLEKHG3	0.9787
GOTERM_MF_FAT	GO:0030695~GTPase regulator activity	4	0.3977	JUN, ALDH1A1, IQSEC3, PLEKHG3	0.9981
GOTERM_MF_FAT	GO:0060589~nucleoside-triphosphatase regulator activity	4	0.4114	JUN, ALDH1A1, IQSEC3, PLEKHG3	0.9974
**Annotation Cluster 21<br>Enrichment Score: 0.4509765462766047**					
**Category**	**Term**	**Count**	**P-Value**	**Genes**	**Benjamini**
GOTERM_BP_FAT	GO:0010605~negative regulation of macromolecule metabolic process	7	0.2573	JUN, STRN3, ADRA1B, BHLHE40, SIK1, PER1, TCF25	0.9828
GOTERM_MF_FAT	GO:0016564~transcription repressor activity	4	0.2622	STRN3, BHLHE40, SIK1, NFIL3	0.9903
GOTERM_BP_FAT	GO:0016481~negative regulation of transcription	5	0.2801	STRN3, BHLHE40, SIK1, PER1, TCF25	0.9868
GOTERM_BP_FAT	GO:0010629~negative regulation of gene expression	5	0.3397	STRN3, BHLHE40, SIK1, PER1, TCF25	0.9880
GOTERM_BP_FAT	GO:0045892~negative regulation of transcription, DNA-dependent	4	0.3450	STRN3, BHLHE40, SIK1, TCF25	0.9850
GOTERM_BP_FAT	GO:0045934~negative regulation of nucleobase, nucleoside, nucleotide and nucleic acid metabolic process	5	0.3504	STRN3, BHLHE40, SIK1, PER1, TCF25	0.9836
GOTERM_BP_FAT	GO:0051253~negative regulation of RNA metabolic process	4	0.3546	STRN3, BHLHE40, SIK1, TCF25	0.9828
GOTERM_BP_FAT	GO:0051172~negative regulation of nitrogen compound metabolic process	5	0.3598	STRN3, BHLHE40, SIK1, PER1, TCF25	0.9832
GOTERM_BP_FAT	GO:0010558~negative regulation of macromolecule	5	0.3972	STRN3, BHLHE40, SIK1, PER1, TCF25	0.9845

	biosynthetic process				
GOTERM_BP_FAT	GO:0031327~negative regulation of cellular biosynthetic process	5	0.4158	STRN3, BHLHE40, SIK1, PER1, TCF25	0.9868
GOTERM_BP_FAT	GO:0006357~regulation of transcription from RNA polymerase II promoter	6	0.4204	KLF9, JUN, STRN3, SIK1, TCF25, NPAS4	0.9863
GOTERM_BP_FAT	GO:0009890~negative regulation of biosynthetic process	5	0.4317	STRN3, BHLHE40, SIK1, PER1, TCF25	0.9865
GOTERM_BP_FAT	GO:0000122~negative regulation of transcription from RNA polymerase II promoter	3	0.4622	STRN3, SIK1, TCF25	0.9898
**Annotation Cluster 22**<br>**Enrichment Score: 0.44742085562098066**					
**Category**	**Term**	**Count**	**P-Value**	**Genes**	**Benjamini**
INTERPRO	IPR000742:EGF-like, type 3	3	0.3036	NOTCH4, PLAT, TENM3	0.9979
INTERPRO	IPR006210:EGF-like	3	0.3183	NOTCH4, PLAT, TENM3	0.9979
SP_PIR_KEYWORDS	Egf-like domain	3	0.3321	NOTCH4, PLAT, TENM3	0.9719
SMART	SM00181:EGF	3	0.3609	NOTCH4, PLAT, TENM3	0.9922
INTERPRO	IPR013032:EGF-like region, conserved site	3	0.5001	NOTCH4, PLAT, TENM3	0.9999
**Annotation Cluster 23**<br>**Enrichment Score: 0.41195696553774774**					
**Category**	**Term**	**Count**	**P-Value**	**Genes**	**Benjamini**
GOTERM_CC_FAT	GO:0005615~extracellular space	6	0.2893	PCSK2, NOTCH4, SCG2, PLAT, CRHBP, LIPE	0.9958
GOTERM_CC_FAT	GO:0044421~extracellular region part	7	0.3898	PCSK2, NOTCH4, SCG2, PLAT, CRHBP, LIPE, DMP1	0.9913
SP_PIR_KEYWORDS	Cleavage on pair of basic residues	3	0.4077	PCSK2, SCG2, PLAT	0.9545
GOTERM_CC_FAT	GO:0005576~extracellular region	12	0.4894	PCSK2, ARTN, NOTCH4, SCG2, RTEL1, PLAT,	0.9921

				PGLYRP1, DSCAM, CRHBP, LIPE, DMP1, PLA1A	

Annotation Cluster 24
Enrichment Score: 0.3578417624010794

Category	Term	Count	P-Value	Genes	Benjamini
GOTERM_BP_FAT	GO:0042981~regulation of apoptosis	7	0.3288	DUSP1, JUN, SCG2, BTG2, RTEL1, MAPK8IP1, FEM1B	0.9882
GOTERM_BP_FAT	GO:0043067~regulation of programmed cell death	7	0.3372	DUSP1, JUN, SCG2, BTG2, RTEL1, MAPK8IP1, FEM1B	0.9883
GOTERM_BP_FAT	GO:0010941~regulation of cell death	7	0.3404	DUSP1, JUN, SCG2, BTG2, RTEL1, MAPK8IP1, FEM1B	0.9873
GOTERM_BP_FAT	GO:0043066~negative regulation of apoptosis	4	0.3418	SCG2, BTG2, RTEL1, MAPK8IP1	0.9859
GOTERM_BP_FAT	GO:0043069~negative regulation of programmed cell death	4	0.3498	SCG2, BTG2, RTEL1, MAPK8IP1	0.9843
GOTERM_BP_FAT	GO:0060548~negative regulation of cell death	4	0.3514	SCG2, BTG2, RTEL1, MAPK8IP1	0.9829
GOTERM_BP_FAT	GO:0043065~positive regulation of apoptosis	3	0.7208	DUSP1, JUN, FEM1B	0.9996
GOTERM_BP_FAT	GO:0043068~positive regulation of programmed cell death	3	0.7244	DUSP1, JUN, FEM1B	0.9996
GOTERM_BP_FAT	GO:0010942~positive regulation of cell death	3	0.7268	DUSP1, JUN, FEM1B	0.9996

Annotation Cluster 25
Enrichment Score: 0.3449708856453159

Category	Term	Count	P-Value	Genes	Benjamini
UP_SEQ_FEATURE	Domain:Ig-like C2-type 1	3	0.2584	CADM4, DSCAM, TYRO3	0.9943
UP_SEQ_FEATURE	Domain:Ig-like C2-type 2	3	0.2603	CADM4, DSCAM, TYRO3	0.9933
INTERPRO	IPR003598:Immunoglobulin subtype 2	3	0.3267	CADM4, DSCAM, TYRO3	0.9975
SMART	SM00408:IGc2	3	0.3700	CADM4, DSCAM, TYRO3	0.9863
SP_PIR_KEYWORDS	Cell adhesion	4	0.3704	CADM4, DSCAM, BAI1, TYRO3	0.9622
GOTERM_BP_FAT	GO:0007155~cell adhesion	6	0.3888	CADM4, CNKSR3, GNE, DSCAM, BAI1, TYRO3	0.9849

GOTERM_BP_FAT	GO:0022610~biological adhesion	6	0.3899	CADM4, CNKSR3, GNE, DSCAM, BAI1, TYRO3	0.9844
INTERPRO	IPR003599:Immunoglobulin subtype	3	0.5643	CADM4, DSCAM, TYRO3	0.9999
SMART	SM00409:IG	3	0.6191	CADM4, DSCAM, TYRO3	0.9981
SP_PIR_KEYWORDS	Immunoglobulin domain	3	0.7002	CADM4, DSCAM, TYRO3	0.9930
INTERPRO	IPR007110:Immunoglobulin-like	3	0.7839	CADM4, DSCAM, TYRO3	1
INTERPRO	IPR013783:Immunoglobulin-like fold	3	0.8283	CADM4, DSCAM, TYRO3	1
**Annotation Cluster 26**<br>**Enrichment Score: 0.3255558100565921**					
**Category**	**Term**	**Count**	**P-Value**	**Genes**	**Benjamini**
GOTERM_BP_FAT	GO:0045893~positive regulation of transcription, DNA-dependent	5	0.3038	TESC, JUN, STRN3, NOTCH4, NPAS4	0.9899
GOTERM_BP_FAT	GO:0051254~positive regulation of RNA metabolic process	5	0.3091	TESC, JUN, STRN3, NOTCH4, NPAS4	0.9893
GOTERM_BP_FAT	GO:0045941~positive regulation of transcription	5	0.4198	TESC, JUN, STRN3, NOTCH4, NPAS4	0.9868
GOTERM_BP_FAT	GO:0006357~regulation of transcription from RNA polymerase II promoter	6	0.4204	KLF9, JUN, STRN3, SIK1, TCF25, NPAS4	0.9863
GOTERM_BP_FAT	GO:0010628~positive regulation of gene expression	5	0.4422	TESC, JUN, STRN3, NOTCH4, NPAS4	0.9872
GOTERM_BP_FAT	GO:0045935~positive regulation of nucleobase, nucleoside, nucleotide and nucleic acid metabolic process	5	0.4977	TESC, JUN, STRN3, NOTCH4, NPAS4	0.9928
GOTERM_BP_FAT	GO:0051173~positive regulation of nitrogen compound metabolic process	5	0.5227	TESC, JUN, STRN3, NOTCH4, NPAS4	0.9948
GOTERM_BP_FAT	GO:0010557~positive regulation of macromolecule biosynthetic process	5	0.5350	TESC, JUN, STRN3, NOTCH4, NPAS4	0.9949

GOTERM_BP_FAT	GO:0010604~positive regulation of macromolecule metabolic process	6	0.5666	TESC, JUN, STRN3, NOTCH4, ADRA1B, NPAS4	0.9965
GOTERM_BP_FAT	GO:0031328~positive regulation of cellular biosynthetic process	5	0.5722	TESC, JUN, STRN3, NOTCH4, NPAS4	0.9963
GOTERM_BP_FAT	GO:0009891~positive regulation of biosynthetic process	5	0.5839	TESC, JUN, STRN3, NOTCH4, NPAS4	0.9967
GOTERM_BP_FAT	GO:0045944~positive regulation of transcription from RNA polymerase II promoter	3	0.6421	JUN, STRN3, NPAS4	0.9984
**Annotation Cluster 27**<br>**Enrichment Score: 0.30183580316266934**					
**Category**	**Term**	**Count**	**P-Value**	**Genes**	**Benjamini**
GOTERM_CC_FAT	GO:0044459~plasma membrane part	15	0.2565	GRIK5, NOTCH4, ADRA1B, NUMB, RIMBP2, LIPE, TYRO3, RTN4RL2, SYT12, LIMK1, CNKSR3, KCNH1, DSCAM, BAI1, ADRM1	1
GOTERM_CC_FAT	GO:0031226~intrinsic to plasma membrane	9	0.3031	NOTCH4, ADRA1B, KCNH1, NUMB, DSCAM, BAI1, TYRO3, ADRM1, RTN4RL2	0.9911
SP_PIR_KEYWORDS	Cell membrane	13	0.4577	GRIK5, PMEPA1, CAMK1G, NOTCH4, ADRA1B, RIMBP2, LIPE, TYRO3, RTN4RL2, GPR162, CNKSR3, BAI1, DSCAM	0.9506
GOTERM_CC_FAT	GO:0005886~plasma membrane	21	0.5246	TESC, GRIK5, PMEPA1, STRN3, NOTCH4, CAMK1G, ADRA1B, NUMB, RIMBP2, LIPE, TYRO3, RTN4RL2, SYT12, GPR162, LIMK1, CNKSR3, KCNH1, DSCAM, BAI1, ADRM1, MARCKSL1	0.9923
SP_PIR_KEYWORDS	Membrane	29	0.8440	PITPNM1, CADM4, CYP46A1, NOTCH4, CAMK1G, COQ7, NUMB, LIPE, TYRO3, RTN4RL2, SYT12,	0.9980

				GPR162, CNKSR3, KCNH1, DSCAM, BAI1, TENM3, GRIK5, HS3ST2, PMEPA1, STRN3, ADRA1B, RIMBP2, CHPF2, SEC61A2, TSPAN2, SLC35F4, COQ10B, KCNK12	
GOTERM_CC_FAT	GO:0031224~intrinsic to membrane	22	0.9808	GRIK5, CADM4, CYP46A1, HS3ST2, PMEPA1, NOTCH4, ADRA1B, NUMB, TYRO3, CHPF2, RTN4RL2, SYT12, SEC61A2, GPR162, TSPAN2, KCNH1, SLC35F4, DSCAM, BAI1, TENM3, ADRM1, KCNK12	1

Annotation Cluster 28
Enrichment Score: 0.29578324848936166

Category	Term	Count	P-Value	Genes	Benjamini
GOTERM_CC_FAT	GO:0031410~cytoplasmic vesicle	5	0.4363	PCSK2, CAMK2G, PLAT, ANXA11, SYT12	0.9922
GOTERM_CC_FAT	GO:0031982~vesicle	5	0.4692	PCSK2, CAMK2G, PLAT, ANXA11, SYT12	0.9918
GOTERM_CC_FAT	GO:0016023~cytoplasmic membrane-bounded vesicle	4	0.5551	PCSK2, CAMK2G, PLAT, ANXA11	0.9906
GOTERM_CC_FAT	GO:0031988~membranebounded vesicle	4	0.5773	PCSK2, CAMK2G, PLAT, ANXA11	0.9915

Annotation Cluster 29
Enrichment Score: 0.24900590625427083

Category	Term	Count	P-Value	Genes	Benjamini
SP_PIR_KEYWORDS	Receptor	10	0.4289	LENG1, GRIK5, GPR162, NOTCH4, ADRA1B, RTEL1, CRY1, BAI1, TYRO3, RTN4RL2	0.9459
SP_PIR_KEYWORDS	Cell membrane	13	0.4577	GRIK5, PMEPA1, CAMK1G, NOTCH4, ADRA1B, RIMBP2, LIPE, TYRO3, RTN4RL2, GPR162, CNKSR3, BAI1, DSCAM	0.9506
UP_SEQ_FEATURE	Topological domain: Extracellular	11	0.9122	CADM4, GRIK5, GPR162, PMEPA1, NOTCH4, ADRA1B, TSPAN2, DSCAM, BAI1, TENM3, TYRO3	1

Annotation Cluster 30<br>Enrichment Score: 0.2306658195974082					
**Category**	**Term**	**Count**	**P-Value**	**Genes**	**Benjamini**
SP_PIR_KEYWORDS	Glycoprotein	25	0.3715	CADM4, PCSK2, NOTCH4, RTEL1, CRHBP, TYRO3, ANXA11, RTN4RL2, GPR162, ARTN, KCNH1, PLAT, PGLYRP1, BAI1, DSCAM, TENM3, GRIK5, HS3ST2, ADRA1B, CHPF2, DMP1, PLA1A, TSPAN2, ADRM1, KCNK12	0.9577
UP_SEQ_FEATURE	Glycosylation site: N-linked (GlcNAc...)	23	0.4798	GRIK5, CADM4, HS3ST2, PCSK2, NOTCH4, ADRA1B, RTEL1, CRHBP, TYRO3, CHPF2, DMP1, RTN4RL2, PLA1A, GPR162, ARTN, TSPAN2, KCNH1, PLAT, PGLYRP1, DSCAM, BAI1, TENM3, KCNK12	0.9999
GOTERM_CC_FAT	GO:0005576~ extracellular region	12	0.4894	PCSK2, ARTN, NOTCH4, SCG2, RTEL1, PLAT, PGLYRP1, DSCAM, CRHBP, LIPE, DMP1, PLA1A	0.9921
UP_SEQ_FEATURE	Disulfide bond	15	0.6112	CADM4, PCSK2, HS3ST2, NOTCH4, ADRA1B, RTEL1, CRHBP, TYRO3, PLA1A, ARTN, PLAT, PGLYRP1, DSCAM, BAI1, TENM3	1
SP_PIR_KEYWORDS	Disulfide bond	15	0.6583	CADM4, PCSK2, HS3ST2, NOTCH4, ADRA1B, RTEL1, CRHBP, TYRO3, PLA1A, ARTN, PLAT, PGLYRP1, DSCAM, BAI1, TENM3	0.9903
SP_PIR_KEYWORDS	Signal	16	0.7188	GRIK5, CADM4, PCSK2, SCG2, NOTCH4, RTEL1, CRHBP, TYRO3, DMP1, RTN4RL2, PLA1A, ARTN, PLAT, PGLYRP1, DSCAM, BAI1	0.9932
UP_SEQ_FEATURE	Signal peptide	16	0.7283	GRIK5, CADM4, PCSK2, SCG2, NOTCH4, RTEL1, CRHBP, TYRO3, DMP1, RTN4RL2, PLA1A, ARTN, PLAT, PGLYRP1, DSCAM, BAI1	1
SP_PIR_KEYWORDS	Secreted	8	0.7769	ARTN, SCG2, RTEL1, PLAT, PGLYRP1, DSCAM, DMP1, PLA1A	0.9965
**Annotation Cluster 31<br>Enrichment Score: 0.21109647410899354**					
**Category**	**Term**	**Count**	**P-Value**	**Genes**	**Benjamini**
GOTERM_CC_FAT	GO:0005654~nucleoplasm	7	0.3156	JUN, STRN3, CIRBP, GNL3, NPAS4,	0.9798

				ANXA11, CREM	
GOTERM_CC_FAT	GO:0031981~nuclear lumen	8	0.6490	JUN, STRN3, CIRBP, GNL3, NPAS4, ANXA11, PAK1IP1, CREM	0.9945
GOTERM_CC_FAT	GO:0070013~intracellular organelle lumen	9	0.7324	MRPL41, JUN, STRN3, CIRBP, GNL3, NPAS4, ANXA11, PAK1IP1, CREM	0.9978
GOTERM_CC_FAT	GO:0043233~organelle lumen	9	0.7562	MRPL41, JUN, STRN3, CIRBP, GNL3, NPAS4, ANXA11, PAK1IP1, CREM	0.9975
GOTERM_CC_FAT	GO:0031974~membrane-enclosed lumen	9	0.7758	MRPL41, JUN, STRN3, CIRBP, GNL3, NPAS4, ANXA11, PAK1IP1, CREM	0.9975

Annotation Cluster 32
Enrichment Score: 0.17834734692956647

**Category**	**Term**	**Count**	**P-Value**	**Genes**	**Benjamini**
GOTERM_BP_FAT	GO:0070271~protein complex biogenesis	4	0.5700	CNKSR3, WASF1, PFKP, ADRM1	0.9965
GOTERM_BP_FAT	GO:0006461~protein complex assembly	4	0.5700	CNKSR3, WASF1, PFKP, ADRM1	0.9965
GOTERM_BP_FAT	GO:0065003~macromolecular complex assembly	4	0.7525	CNKSR3, WASF1, PFKP, ADRM1	0.9997
GOTERM_BP_FAT	GO:0043933~macromolecular complex subunit organization	4	0.7912	CNKSR3, WASF1, PFKP, ADRM1	0.9999

Annotation Cluster 33
Enrichment Score: 0.1668762657503625

**Category**	**Term**	**Count**	**P-Value**	**Genes**	**Benjamini**
SP_PIR_KEYWORDS	ionic channel	3	0.4890	GRIK5, KCNH1, KCNK12	0.9562
GOTERM_MF_FAT	GO:0022836~gated channel activity	3	0.5234	GRIK5, KCNH1, KCNK12	0.9994
GOTERM_MF_FAT	GO:0005216~ion channel activity	3	0.6433	GRIK5, KCNH1, KCNK12	0.9998
GOTERM_MF_FAT	GO:0022838~substrate specific channel activity	3	0.6600	GRIK5, KCNH1, KCNK12	0.9998
GOTERM_MF_FAT	GO:0015267~channel activity	3	0.6786	GRIK5, KCNH1, KCNK12	0.9998

GOTERM_MF_FAT	GO:0022803~passive transmembrane transporter activity	3	0.6799	GRIK5, KCNH1, KCNK12	0.9997
SP_PIR_KEYWORDS	Ion transport	3	0.8024	GRIK5, KCNH1, KCNK12	0.9968
GOTERM_BP_FAT	GO:0006811~ion transport	4	0.8337	GRIK5, CAMK2G, KCNH1, KCNK12	1
SP_PIR_KEYWORDS	Transport	6	0.9387	GRIK5, SEC61A2, PITPNM1, SLC35F4, KCNH1, KCNK12	0.9999

Annotation Cluster 34
Enrichment Score: 0.1407674184058494

Category	Term	Count	P-Value	Genes	Benjamini
GOTERM_CC_FAT	GO:0005739~mitochondrion	6	0.6842	MRPL41, COQ7, CRY1, COQ10B, MRPS34, WASF1	0.9960
UP_SEQ_FEATURE	Transit peptide: Mitochondrion	3	0.7002	MRPL41, COQ7, COQ10B	1
SP_PIR_KEYWORDS	Transit peptide	3	0.7069	MRPL41, COQ7, COQ10B	0.9928
SP_PIR_KEYWORDS	Mitochondrion	4	0.8075	MRPL41, COQ7, COQ10B, MRPS34	0.9967

Annotation Cluster 35
Enrichment Score: 0.13343210896498828

Category	Term	Count	P-Value	Genes	Benjamini
GOTERM_CC_FAT	GO:0015630~microtubule cytoskeleton	4	0.5539	KIFC2, MARK1, ANXA11, RASSF5	0.9921
GOTERM_CC_FAT	GO:0005856~cytoskeleton	7	0.7447	KIFC2, MARK1, GNE, WASF1, ANXA11, RASSF5, SORBS2	0.9974
GOTERM_CC_FAT	GO:0044430~cytoskeletal part	3	0.9646	KIFC2, ANXA11, RASSF5	1

Annotation Cluster 36
Enrichment Score: 0.13243049963651077

Category	Term	Count	P-Value	Genes	Benjamini
GOTERM_CC_FAT	GO:0043228~non-membrane-bounded organelle	13	0.7335	MRPL41, JUN, FXR2, KIFC2, MARK1, MRPS34, GNE, WASF1, GNL3, ANXA11, RASSF5, SORBS2, PAK1IP1	0.9974

GOTERM_CC_FAT	GO:0043232~intracellular non-membrane-bounded organelle	13	0.7335	MRPL41, JUN, FXR2, KIFC2, MARK1, MRPS34, GNE, WASF1, GNL3, ANXA11, RASSF5, SORBS2, PAK1IP1	0.9974
GOTERM_CC_FAT	GO:0005856~cytoskeleton	7	0.7447	KIFC2, MARK1, GNE, WASF1, ANXA11, RASSF5, SORBS2	0.9974
**Annotation Cluster 37**<br>**Enrichment Score: 0.11824527499037826**					
**Category**	**Term**	**Count**	**P-Value**	**Genes**	**Benjamini**
SP_PIR_KEYWORDS	Glycoprotein	25	0.3715	CADM4, PCSK2, NOTCH4, RTEL1, CRHBP, TYRO3, ANXA11, RTN4RL2, GPR162, ARTN, KCNH1, PLAT, PGLYRP1, BAI1, DSCAM, TENM3, GRIK5, HS3ST2, ADRA1B, CHPF2, DMP1, PLA1A, TSPAN2, ADRM1, KCNK12	0.9577
UP_SEQ_FEATURE	Glycosylation site:N-linked (GlcNAc...)	23	0.4798	GRIK5, CADM4, HS3ST2, PCSK2, NOTCH4, ADRA1B, RTEL1, CRHBP, TYRO3, CHPF2, DMP1, RTN4RL2, PLA1A, GPR162, ARTN, TSPAN2, KCNH1, PLAT, PGLYRP1, DSCAM, BAI1, TENM3, KCNK12	0.9999
UP_SEQ_FEATURE	Topological domain: Cytoplasmic	17	0.6918	GRIK5, CADM4, PMEPA1, HS3ST2, NOTCH4, ADRA1B, TYRO3, CHPF2, SYT12, GPR162, SEC61A2, TSPAN2, KCNH1, DSCAM, BAI1, TENM3, KCNK12	1
SP_PIR_KEYWORDS	Membrane	29	0.8440	PITPNM1, CADM4, CYP46A1, NOTCH4, CAMK1G, COQ7, NUMB, LIPE, TYRO3, RTN4RL2, SYT12, GPR162, CNKSR3, KCNH1, DSCAM, BAI1, TENM3, GRIK5, HS3ST2, PMEPA1, STRN3, ADRA1B, RIMBP2, CHPF2, SEC61A2, TSPAN2, SLC35F4, COQ10B, KCNK12	0.9980
UP_SEQ_FEATURE	Topological domain: Extracellular	11	0.9122	CADM4, GRIK5, GPR162, PMEPA1, NOTCH4, ADRA1B, TSPAN2, DSCAM, BAI1, TENM3, TYRO3	1
UP_SEQ_FEATURE	Transmembrane	19	0.9702	GRIK5, CADM4, CYP46A1, PMEPA1, HS3ST2, NOTCH4,	1

	region			ADRA1B, TYRO3, CHPF2, SYT12, SEC61A2, GPR162, TSPAN2, KCNH1, SLC35F4, DSCAM, BAI1, TENM3, KCNK12	
SP_PIR_KEYWORDS	Transmembrane	19	0.9725	GRIK5, CADM4, CYP46A1, PMEPA1, HS3ST2, NOTCH4, ADRA1B, TYRO3, CHPF2, SYT12, SEC61A2, GPR162, TSPAN2, KCNH1, SLC35F4, DSCAM, BAI1, TENM3, KCNK12	1
GOTERM_CC_FAT	GO:0031224~intrinsic to membrane	22	0.9808	GRIK5, CADM4, CYP46A1, HS3ST2, PMEPA1, NOTCH4, ADRA1B, NUMB, TYRO3, CHPF2, RTN4RL2, SYT12, SEC61A2, GPR162, TSPAN2, KCNH1, SLC35F4, DSCAM, BAI1, TENM3, ADRM1, KCNK12	1
GOTERM_CC_FAT	GO:0016021~integral to membrane	21	0.9816	GRIK5, CADM4, CYP46A1, PMEPA1, HS3ST2, NOTCH4, ADRA1B, NUMB, TYRO3, CHPF2, SYT12, SEC61A2, GPR162, TSPAN2, KCNH1, SLC35F4, DSCAM, BAI1, TENM3, ADRM1, KCNK12	1

Annotation Cluster 38
Enrichment Score: 0.09134641011708738

Category	Term	Count	P-Value	Genes	Benjamini
GOTERM_BP_FAT	GO:0030001~metal ion transport	3	0.7604	CAMK2G, KCNH1, KCNK12	0.9998
GOTERM_BP_FAT	GO:0006811~ion transport	4	0.8337	GRIK5, CAMK2G, KCNH1, KCNK12	1
GOTERM_BP_FAT	GO:0006812~cation transport	3	0.8393	CAMK2G, KCNH1, KCNK12	1

Annotation Cluster 39
Enrichment Score: 0.0691244064620221

Category	Term	Count	P-Value	Genes	Benjamini
GOTERM_BP_FAT	GO:0015031~protein transport	4	0.8297	JUN, SEC61A2, PITPNM1, SCG2	1
GOTERM_BP_FAT	GO:0045184~establishment of protein localization	4	0.8343	JUN, SEC61A2, PITPNM1, SCG2	1
GOTERM_BP_FAT	GO:0008104~protein localization	4	0.8962	JUN, SEC61A2, PITPNM1, SCG2	1

**Annotation Cluster 40**<br>**Enrichment Score: 0.06420315397565153**					
**Category**	**Term**	**Count**	**P-Value**	**Genes**	**Benjamini**
SP_PIR_KEYWORDS	Metal-binding	15	0.6830	PDE4B, KLF9, PITPNM1, CYP46A1, COQ7, RTEL1, MARK1, MAP3K6, RASSF5, ZMYND8, LIMK1, PHRF1, ASPA, SIK1, PFKP	0.9921
GOTERM_MF_FAT	GO:0046872~metal ion binding	22	0.7554	TESC, PITPNM1, KLF9, CYP46A1, SCG2, NOTCH4, COQ7, RTEL1, MARK1, ANXA11, RASSF5, DMP1, MAP3K6, ZMYND8, LIMK1, PHRF1, ASPA, KCNH1, SIK1, PFKP, SORBS2, KCNK12	0.9999
GOTERM_MF_FAT	GO:0043169~cation binding	22	0.7722	TESC, PITPNM1, KLF9, CYP46A1, SCG2, NOTCH4, COQ7, RTEL1, MARK1, ANXA11, RASSF5, DMP1, MAP3K6, ZMYND8, LIMK1, PHRF1, ASPA, KCNH1, SIK1, PFKP, SORBS2, KCNK12	0.9999
GOTERM_MF_FAT	GO:0043167~ion binding	22	0.7975	TESC, PITPNM1, KLF9, CYP46A1, SCG2, NOTCH4, COQ7, RTEL1, MARK1, ANXA11, RASSF5, DMP1, MAP3K6, ZMYND8, LIMK1, PHRF1, ASPA, KCNH1, SIK1, PFKP, SORBS2, KCNK12	0.9999
GOTERM_MF_FAT	GO:0046914~transition metal ion binding	10	0.9847	ZMYND8, KLF9, CYP46A1, LIMK1, COQ7, PHRF1, RTEL1, ASPA, RASSF5, SORBS2	1
SP_PIR_KEYWORDS	Zinc	6	0.9909	ZMYND8, KLF9, LIMK1, PHRF1, ASPA, RASSF5	1
GOTERM_MF_FAT	GO:0008270~zinc ion binding	7	0.9938	ZMYND8, KLF9, LIMK1, PHRF1, ASPA, RASSF5, SORBS2	1
SP_PIR_KEYWORDS	Zinc-finger	4	0.9946	ZMYND8, KLF9, PHRF1, RASSF5	1

**Annotation Cluster 41**<br>**Enrichment Score: 0.023405171688667542**					
**Category**	**Term**	**Count**	**P-Value**	**Genes**	**Benjamini**
SP_PIR_KEYWORDS	G-protein coupled receptor	3	0.9268	GPR162, ADRA1B, BAI1	0.9998
GOTERM_BP_FAT	GO:0007166~cell surface receptor linked signal transduction	8	0.9300	JUN, GPR162, NOTCH4, CNKSR3, ADRA1B, PLAT, BAI1, TLE3	1
SP_PIR_KEYWORDS	Transducer	3	0.9432	GPR162, ADRA1B, BAI1	0.9999
GOTERM_BP_FAT	GO:0007186~G-protein coupled receptor protein signaling pathway	3	0.99151	GPR162, ADRA1B, BAI1	1
